# The topology of the magnetically induced ring current of C_13_Cl_2_

**DOI:** 10.1039/d6sc03398a

**Published:** 2026-06-27

**Authors:** Qian Wang, Rinat T. Nasibullin, Glib Baryshnikov, Sophia Burger, Jürgen Gauss, Rashid R. Valiev, Dage Sundholm

**Affiliations:** a Department of Chemistry, Faculty of Science, University of Helsinki P.O. Box 55tbox55 (A.I. Virtanens plats 1) FIN-00014 Finland Dage.Sundholm@helsinki.fi; b Laboratory of Organic Electronics, Department of Science and Technology, Linköping University SE-601 74 Norrköping Sweden; c Department Chemie, Johannes Gutenberg-Universität Mainz Duesbergweg 10-14 D-55128 Mainz Germany

## Abstract

We have performed density functional theory (DFT), second-order Møller–Plesset perturbation theory (MP2), coupled cluster singles and doubles (CCSD), and complete-active-space self-consistent-field (CASSCF) calculations of the magnetically induced current density (MICD) susceptibility of the molecular structures of C_13_Cl_2_ belonging to the *C*_2_ and *C*_2v_ point groups. The ring of the planar *C*_2v_ structure of C_13_Cl_2_ is aromatic sustaining a strong diatropic magnetically induced ring current (MIRC) and no paratropic contribution to the MIRC. The paratropic MIRC of the *C*_2_ structure suggests that it is weakly antiaromatic. Its paratropic MIRC makes several vertical loops around the cumulene part of the ring and passes on the inside of the other half of the ring. The strongest MIRC trajectories have a very unusual topology with a very large linking number (*L*_k_) of 6π, which is the same as we obtained for the helical highest-occupied molecular orbital (HOMO). Our study shows that it is not necessary to introduce the half-Möbius topology concept to understand the electronic structure and the magnetic response of the *C*_2_ structure of C_13_Cl_2_.

## Abstract 2

The strongest ring-current streamline and the highest-occupied molecular orbital of C_13_Cl_2_ have a topology linking number of 6π.

## Graphical abstract

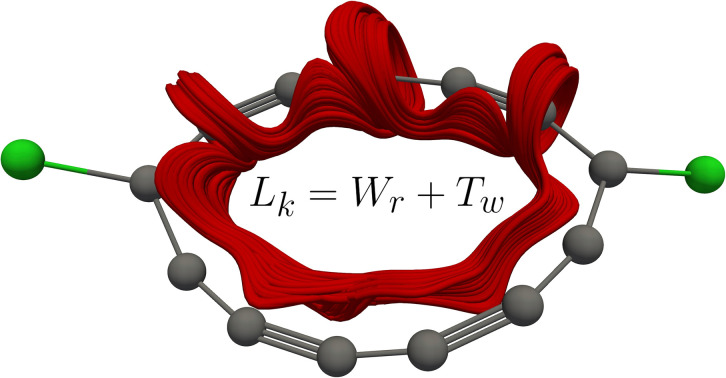

## 1 Introduction

Rončević *et al.*^1^ have recently reported the synthesis of C_13_Cl_2_ on a NaCl surface together with its characterization. The C_13_Cl_2_ ring consisting of thirteen carbon atoms is expected to have very similar properties on the surface as in the gas phase because the interaction with the NaCl surface is very weak.^1^ Since the Cl atoms are attached to the sp^2^-hybridized C_1_ and C_7_ atoms, the eleven sp-hybridized carbon atoms are divided into two segments, one of which has seven carbon–carbon bonds (eight atoms) and the other segment has six carbon–carbon bonds (seven atoms). Cumulene segments consisting of an odd number of carbon atoms have perpendicular end groups leading to helical frontier orbitals,^2–5^ whereas cumulene segments with an even number of carbon atoms have coplanar end groups and Hückel-type π orbitals.^3^ Connecting the two segments to form a ring leads to a 90° (π/2 radian) rotation of the p orbitals around the ring.^1,3^ Thus, following the ring frame one lap around the ring rotates the positive lobe of the vertical p orbital to the position of *e.g.* the positive lobe of the horizontal p orbital. The second lap rotates it to the position of the negative lobe of the vertical p orbital. Thus, four laps are needed to reach the starting point. Since this is twice the number of laps as compared to single Möbius-twisted molecules, the authors of ref. 1 called it half-Möbius topology. This holds for the chiral C_13_Cl_2_ ring whose molecular structure belongs to the *C*_2_ point group. The molecular structure belonging to the *C*_2v_ point group is the transition state between the two chiral *C*_2_ structures on the potential energy surface of the lowest singlet state. The molecular structure of the lowest triplet state is symmetry broken belonging to the *C*_1_ point group.^1^

The Călugăreanu–White–Fuller theorem describes the topology of closed ribbons^6–9^ implying that chemical applications require that molecular properties are mapped onto a ribbon. A ribbon representing the molecular structure is constructed using the cross product of the vectors along adjacent carbon–carbon bonds. The topology of the frontier orbitals is obtained by constructing a ribbon whose edges follow the largest positive or negative amplitude of the studied orbital. A large number of ribbons representing the magnetically induced ring current (MIRC) is obtained by studying pairs of MIRC trajectories that were generated using the Runge–Kutta integration scheme.^10,11^ Topological properties of conjugated molecular rings containing sp^2^- and sp- hybridized carbon atoms have been previously analyzed using the Călugăreanu–White–Fuller theorem,^12–15^ which to our knowledge has not been generalized to hold for four-edged ribbons as introduced in ref. 1.

Here, we compute and analyze the magnetically induced current density (MICD) susceptibility of C_13_Cl_2_ using the gauge-including magnetically induced currents (GIMIC) method.^16–18^ The calculations show that completely different MICD susceptibilities are sustained by the *C*_2_ and *C*_2v_ structures of C_13_Cl_2_.

## 2 Computational methods

The molecular structures of C_13_Cl_2_ belonging to the *C*_2_ and *C*_2v_ point groups were optimized with Turbomole version 7.9 (ref. 19–21) at the density functional theory (DFT) level using the ωB97X functional,^22^ the empirical D4 dispersion correction,^23^ and the def2-TZVP basis sets.^24^ The molecular structure of the lowest triplet state was optimized at the same level of theory without any symmetry constraints (*C*_1_ point group). Calculations of the vibrational frequencies showed that the *C*_2_ structure is the minimum, whereas the *C*_2v_ structure is the transition state between the two chiral *C*_2_ minima. The lowest excitation energies were calculated at the approximate second-order coupled cluster level (CC2),^25,26^ at the coupled-cluster singles and doubles (CCSD) response theory level,^27^ and at the time-dependent DFT (TDDFT)^28,29^ level. The total energies of the *C*_2_ and *C*_2v_ structures were also calculated in a more reliable manner at the CCSD level^30^ and at the CCSD level with a perturbative treatment of the triples (CCSD(T)).^31,32^ The geometry of the *C*_2_ structure was optimized at the CCSD/def2-TZVP level with the ccsdf12 program^32^ of Turbomole using numerical gradients.

Calculations at the complete-active-space self-consistent-field (CASSCF) level^33^ with 12 electrons in 12 active orbitals (12,12) showed that for the *C*_2_ structure, the ground-state wave function is dominated by one Slater determinant. The rest of the wave function consists of small contributions from many Slater determinants suggesting that it is a strongly correlated wave function dominated by one Slater determinant. Increasing the size of the active space accounts not only for static correlation but also for an increasing amount of dynamic correlation. However, this comes at the price of rapidly increasing computational costs. Today's DFT functionals perform well when dynamic correlation effects are significant.^34^ The wave function can also be reasonably well described at coupled-cluster levels of theory, which account for dynamic correlation effects. However, when the molecular structure is displaced from its equilibrium geometry, the weights of other configurations than the dominating one increase. When using the molecular structure optimized at the ωB97X level, which significantly differs from the CASSCF equilibrium structure (see the bond-length table in the SI), the coefficient of the dominant configuration obtained using the (12,12) active space drops to 0.78. The configuration coefficient of the dominating configuration becomes smaller when increasing the size of the active space, which suggests that lots of configurations contribute to the wave function, whereas only one configuration dominates implying that single reference methods such as CCSD are accurate.

We also used the mixed-reference spin-flip time-dependent DFT (MRSF-TDDFT) method for optimizing the molecular structure of the ground-state.^35,36^ The MRSF-TDDFT calculations were performed with GAMESS-US^37^ using the BHLYP functional^38^ and the cc-pVDZ basis set.^39,40^ The MRSF-TDDFT calculations show that the ground state is a singlet, whose molecular structure belongs to the *C*_2_ point group. The ground state is dominated by one main determinant whose coefficient is 0.93 (corresponding to 87% of the electron density), while the remaining important configurations have coefficients of +0.26 (6.6%), −0.18 (3.2%), −0.07 (0.5%), and −0.06 (0.3%) indicating that the wave function has a single-reference character.

We investigated the multireference character further by performing calculations using the extended multi-state version of the complete-active-space second-order perturbation theory (XMS-CASPT2) method with BAGEL.^41,42^ Furthermore, excitation energies were calculated at the XMS-CASPT2 level. The active space consisted of 8 electrons in 8 orbitals, which yielded a rapid convergence of the wave function and geometry optimizations without oscillatory gradient behavior. The optimized ground state at the XMS-CASPT2 level is a singlet whose molecular structure belongs to the *C*_2_ point group. The ground state is dominated by one determinant whose coefficient is 0.93 (86%), while the coefficients of the next two determinants are 0.15 (2.3%) and 0.15 (2.2%), respectively.

Nuclear magnetic resonance (NMR) shielding tensors were calculated with Turbomole^43–47^ at the DFT level using the ωB97X functional and at second-order Møller–Plesset perturbation theory (MP2) level using the def2-TZVP basis sets.^24^ The NMR shielding tensors were also calculated at the MP2 and CCSD levels using the cc-pVDZ basis sets^39,40^ and the frozen-core approximation with the CFOUR program package.^48,49^ In addition, we performed CASSCF/cc-pVDZ calculation for the shielding tensors^50,51^ using the CFOUR program package for various active spaces. Cholesky decomposition of the unperturbed and perturbed two-electron integrals with a Cholesky threshold of 10^−10^ was used to speed up the CASSCF computations.^51,52^ Gauge-including atomic orbitals (GIAO) were used in the calculations of the NMR shielding tensors.^53–55^ While for the *C*_2_ structure, the CCSD and the CASSCF shieldings qualitatively agree, the CASSCF computations for the *C*_2v_ structure do not provide convergence with respect to the active space, that is, not even a qualitative agreement is obtained between the NMR shielding tensors calculated at the CASSCF and CCSD levels. The MP2 results are, as expected, not reliable and thus will not be discussed further.

Gauge-origin independent magnetically induced current density (MICD) susceptibilities were calculated with the GIMIC program using the one-electron density matrix and their magnetically perturbed counterparts in the atomic orbital basis obtained in the NMR shielding calculations. The MICD is obtained by contracting the MICD susceptibility tensor with an external magnetic field of a given direction. The Cartesian coordinates of the molecular structures and the basis-set information are also needed as input data for the GIMIC calculations.^16–18,56,57^ The magnetically induced current density (MICD) was obtained by contracting the MICD susceptibility with an external magnetic field that was applied perpendicularly to the molecular ring.^16–18,56,57^ The MICD was separated into diatropic and paratropic contributions using a Runge–Kutta algorithm.^58^ The MICD is visualized using Paraview.^59^

The strength of the magnetically induced ring current (MIRC) was obtained by using the integral form of the Ampère–Maxwell law.^60^ The strength of the MIRC passing through a surface (**S**) was obtained by integrating the induced magnetic field along the edge around the surface. A two-dimensional integration of the MICD (**J**) passing through **S** also yielded the MIRC strength. The strength of the MIRC (*I*) was calculated using1
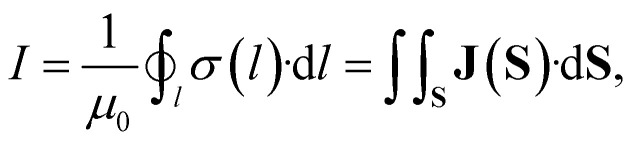
where *σ*(*l*) contains the NMR shielding tensor elements for a given direction of the external magnetic field, *l* is the edge of the surface and and *µ*_0_ = 1.25663706212(19) × 10^−6^ N A^−2^ is the vacuum permeability. Here, the *σ*_*yy*_(*l*) values are calculated along the *y* axis which is perpendicular to the molecular ring. The MIRC strength is obtained by integrating the *σ*_*yy*_(*l*) values from the center of the molecular ring to very far away from the molecule in both directions, where the induced magnetic field vanishes. Orbital contributions to the MIRC were also calculated to identify orbitals sustaining the MIRC.^61^

The aromatic nature can be determined from the direction of the MIRC or using other criteria.^62^ Aromatic molecules have, *e.g.*, a smaller bond-length alternation than antiaromatic and nonaromatic molecules.^63^ Molecules sustaining a net diatropic (in the classical direction) MIRC are aromatic, whereas in antiaromatic molecules the MIRC circulates in the paratropic (the opposite) direction.^16,17,57,64^

### 2.1 Möbius topology

The Călugăreanu–White–Fuller theorem states that Möbius topology is characterized by the linking number (*L*_k_), which is the sum (*L*_k_ = *W*_r_ + *T*_w_) of the writhe (*W*_r_), *i.e.*, the global deformation of the ribbon and its local twist (*T*_w_).^6–9,12^*L*_k_ is a positive or negative integer multiplied by π radians, which is often omitted in discussions. We will use this convention in the following as well. *T*_w_ and *W*_r_ are real numbers that depend on the local twist and deformation of the ribbon.

**Fig. 1:**
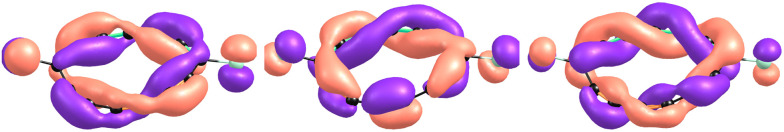
The helical HOMO-4 (left), HOMO-3 (middle) and HOMO (right) of the *C*_2_ structure of C_13_Cl_2_. The rest of the orbitals are shown in the SI.

The topology of conjugated cyclic molecules can be represented by a ribbon defined by the molecular backbone as the centerline with the local orientation of the p orbitals describing the edges.^12^ The topology of the molecular ring is then characterized by *L*_k_, *T*_w_ and *W*_r_. Defining the local orientation of the p orbitals of C_13_Cl_2_ is complicated because molecules with sp-hybridized carbon atoms have horizontal and vertical p orbitals and the highest-occupied molecular orbital (HOMO) is helical (see Fig. 1). MIRC pathways also provide information about the effective topology of the conjugated molecular ring. Since the MIRC has contributions from many occupied orbitals, the MIRC pathway does not necessarily follow the contour of the frontier orbitals.

The MICD calculated with GIMIC is a vector field discretized on a numerical grid. The MICD has both local and global MICD vortices whose topologies differ. To analyze the topology of the dominating MIRC, many streamlines associated with the strongest global MIRC pathway were generated using the Runge–Kutta integration scheme implemented in ParaView.^59^ The obtained streamlines were examined pairwise when assuming an even *L*_k_ and three streamlines are needed when *L*_k_ is odd. When *L*_k_ is odd, an additional streamline was constructed exactly midway between the two streamlines to ensure that the ribbon is defined by three streamlines. Each pair or triple of streamlines forms a ribbon for calculating *L*_k_, *T*_w_, and *W*_r_ using the Rappaport–Rzepa approach.^12,65^ Different *T*_w_ and *W*_r_ values were obtained for each streamline pair (triple), whereas *L*_k_ is constant. The *T*_w_ and *W*_r_ values were therefore averaged over the entire set of pairs, whereas the *L*_k_ value is the same integer when the computational procedure works and the streamlines represent the same MIRC pathway.

The applicability of the procedure was verified by performing calculations on Hückel-aromatic benzene and on the Möbius-aromatic C_9_H_9_^+^ ring.^66^ The generated streamlines for benzene, C_9_H_9_^+^ and C_13_Cl_2_ are shown in the SI.

For benzene, the *L*_k_ value and the average of the *T*_w_, and *W*_r_ values of the MIRC are all zero, which is in agreement with the topology of the molecular ring. For C_9_H_9_^+^ whose molecular structure has *L*_k_ = 1, we obtained average *T*_w_ and *W*_r_ values of −0.51 and 1.51 for the MIRC, whereas the *T*_w_ and *W*_r_ values of the molecular ring are 1.36 and −0.36, respectively. The *T*_w_ and *W*_r_ values of the molecular ring and of the streamline triples of the MIRC differ because the MIRC streamlines do not follow the molecular frame. However, the molecular structure and the MIRC have the same *L*_k_ value and thus the same topology.

## 3 Results and discussion

### 3.1 Molecular structures

Rončević *et al.*^1^ state that the p orbitals of the molecular ring are twisted 90° after one lap and 180° after two laps. Thus, four laps are needed to reach the starting point. According to the Călugăreanu–White–Fuller theorem, *L*_k_ is a positive or negative integer. Thus, half-Möbius 
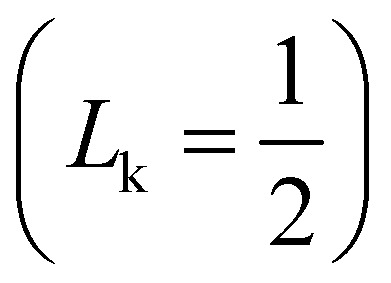
 topology is not supported by the theorem.^6–9^

The topology can be scrutinized by cutting a narrow paper ribbon and marking its sides. Wrapping the ribbon four laps around a cylinder and twisting the end by 2π before connecting the ends simulates the topology described by Rončević *et al.*^1^ Then, one notices that the ribbon has two sides, implying that *L*_k_ is even.^12,67^ When the ribbon is wrapped around a cylinder, each turn changes *L*_k_ by ±2. Depending on how the ribbon is wrapped around the cylinder and how it is twisted, it has a Möbius-twisted topology with |*L*_k_| = 4 or |*L*_k_| = 8 after four laps around the cylinder and a 360° twist of the ribbon. Thus, it is not necessary to introduce half-Möbius topology but the topology of the electronic structure of the *C*_2_ structure of C_13_Cl_2_ can be understood using the Călugăreanu–White–Fuller theorem. Topological properties of Möbius-twisted molecules have been discussed in review articles.^68,69^

**Fig. 2:**
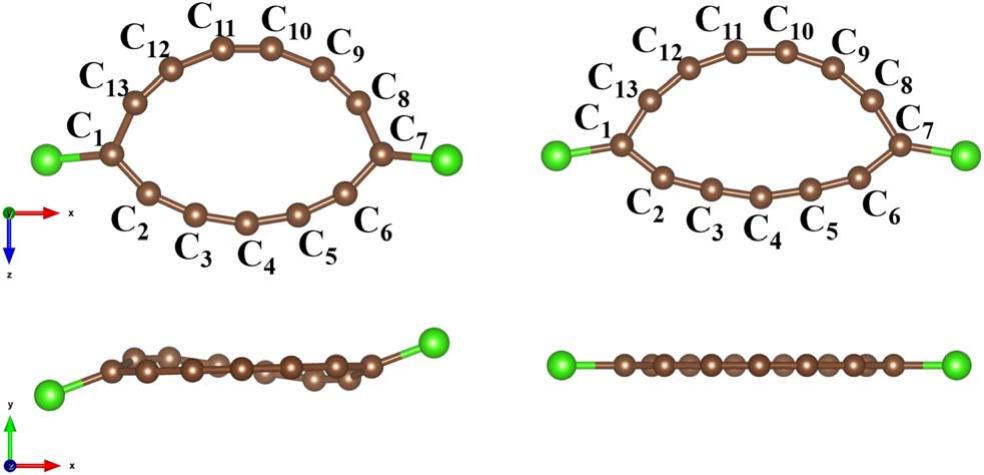
The molecular structures of C_13_Cl_2_ belonging to the *C*_2_ (left) and *C*_2v_ (right) point groups.

The Cartesian coordinates of the molecular structures optimized at the ωB97X/D4/def2-TZVP level are given in the SI. The *C*_2_ axis passes through the molecular ring at atom C_4_ and between the C_10_ and C_11_ atoms. See Fig. 2. The *y* axis is perpendicular to the ring. The *C*_2_ structure is chiral because it does not have inversion symmetry. The calculated *L*_k_ value of the *C*_2_ structure is zero suggesting that the molecular structure is not twisted but has Hückel topology. The *L*_k_ value was obtained by defining a ribbon using the cross product of the vectors that describe the chemical bonds between the carbon atoms.

Substituting Cl atoms to the C_1_ and C_7_ atoms of the *C*_2_ structure leads to a long C–C distance of 1.419 Å between C_1_ (C_7_) and C_13_ (C_8_). The other C–C distance of the sp^2^ hybridized C_1_ (C_7_) atom is much shorter (1.327 Å). The rest of the bond distances along the shorter segment are almost equal leading to a cumulene structure. The distances are 1.267 Å and 1.281 Å. The bond distances along the longer segment alternate. They are 1.211 Å, 1.367 Å  and 1.212 Å. The optimized molecular structure at the CCSD/def2-TZVP level has about 1 pm longer bond lengths than the one obtained at the ωB97X/D4 level with an almost identical bond-length alternation. The bond lengths are compared in the SI.

The sp^2^ hybridized carbon atoms of the *C*_2v_ structure have almost equal C–C distances of 1.377 Å and 1.347 Å. The shorter segment has a cumulene structure with bond distances of 1.261 Å and 1.282 Å for the rest of the bonds. The bond lengths along the longer segment alternate less than for the *C*_2_ structure. They are 1.232 Å, 1.327 Å  and 1.232 Å. The smaller bond-length alternation suggests that the *C*_2v_ structure of C_13_Cl_2_ is more aromatic than the *C*_2_ one.^63^ The *C*_2v_ structure is planar, which means that *L*_k_ = *T*_w_ = *W*_r_ = 0. Note that ribbons with *L*_k_ = 0 can have *W*_r_ = −*T*_w_ with |*W*_r_| = |*T*_w_| ≠ 0.

The *C*_2_ structure is the global minimum at the Hartree–Fock self-consistent-field (HF-SCF) and ωB97X/D4 levels, whereas at the MP2 level, the *C*_2v_ structure has the lowest energy. Coupled-cluster calculations at the CCSD level yielded the lowest energy for the *C*_2_ structure, which was confirmed at the CCSD(T) level. The relative energies (in kJ mol^−1^) between the *C*_2_ and *C*_2v_ structures are given in Table 1. Multireference calculations also showed that the *C*_2_ structure is the energetically lowest one.

**Table 1:** The relative energy (in kJ mol^−1^) between the *C*_2_ and *C*_2v_ structures of C_13_Cl_2_. The molecular structures were optimized at the ωB97X/D4 level. Positive values mean that the *C*_2_ structure is energetically below the *C*_2v_ one

HF-SCF	ωB97X/D4	MP2	CCSD	CCSD(T)
51.2	23.7	−66.4	48.7	20.1

CC2 calculations of excitation energies showed that the triplet state is significantly above the singlet state for both molecular structures, whereas the singlet state of the *C*_2_ structure suffers from a triplet instability at the ωB97X/D4 level. The excitation energies calculated at the CC2 level are given in Table 2. Calculations at the CCSD(T) level for the lowest triplet states showed that the singlet state is 81 kJ mol^−1^ (172 kJ mol^−1^) below the triplet state for the *C*_2_ (*C*_2v_) structure.

**Table 2:** The lowest excitation energies (in eV) of each irreducible representation of the *C*_2_ and *C*_2v_ structures of C_13_Cl_2_ calculated at the CC2/def2-TZVP level

*C*_2_ structure			*C*_2v_ structure		
State	Singlet^a^	Triplet	State	Singlet^b^	Triplet
A	1.163	0.576	A_1_	2.215	2.110
B	1.560	1.266	A_2_	0.633	0.670
			B_1_	3.257	2.625
			B_2_	1.125	0.936

a The excitation energies (in eV) of the lowest singlet states calculated at the CCSD level are 1.581 (A), 1.956 (B).

b The excitation energies at the CCSD level are 2.701 (A_1_), 1.196 (A_2_), 3.157 (B_1_), and 0.986 (B_2_).

We performed single-point calculations of the electronic excitation energies at the XMS-CASPT2(8,8) level using the *C*_2_ and *C*_2v_ structures as well as the optimized molecular structure of the lowest triplet state (T_1_) state. The multi-state averaging was performed in separate calculations over four states in the singlet and triplet manifolds. The XMS-CASPT2 calculations suggest that the closed-shell singlet state is the ground state for all molecular structures studied. The excitation energies are reported in the SI.

### 3.2 Orbital contributions to the MIRC strength

**Fig. 3:**
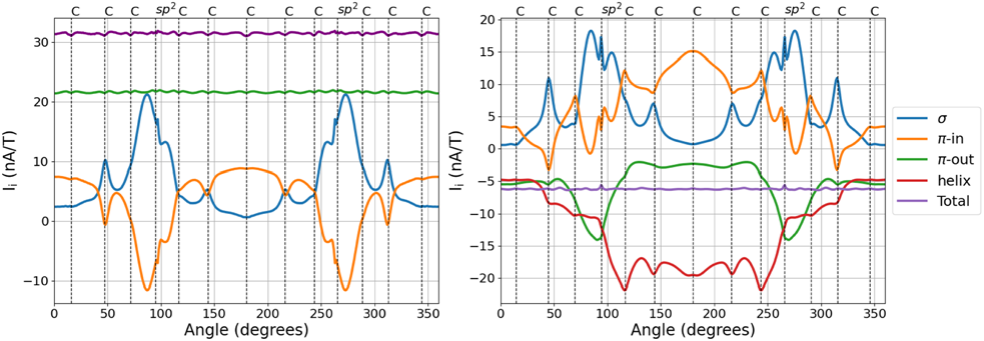
The angular dependence of the total MIRC strength of C_13_Cl_2_ and contributions to the MIRC from core, valence σ, π-in, π-out and helical orbitals for *C*_2v_ (left) and *C*_2_ (right) structures. The vertical line on the plots represents the angle of rotation of the integration plane as it passes through the atomic nucleus. The carbon atom bonded to chlorine is denoted as sp^2^. The central part of both plots between the sp^2^ atoms corresponds to the cumulenic fragment, while the remaining part corresponds to the polyenic.

The molecular orbitals of the planar *C*_2v_ structure of C_13_Cl_2_ can be divided into three groups: σ, π-in, and π-out. However, due to the presence of the C–Cl bonds, the π-in and σ orbitals are mixed near the sp^2^ carbons, which is evident from the angular dependence of the orbital contributions to the MIRC strength shown in Fig. 3. The contribution of the π-out orbitals is almost constant, indicating that these orbitals do not mix with the other ones. The total diatropic MIRC strength of 31.5 nA T^−1^ is mainly sustained by the π-out orbitals whose contribution is 21.7 nA T^−1^. See Table 3. The σ-orbital contribution is 6.4 nA T^−1^, indicating that the molecular structure is strained. None of the orbitals has a dominant MIRC contribution within the orbital decomposition approach used here. Instead, the MIRC strength is distributed over several occupied orbitals, as previously observed for other aromatic molecules analyzed using the same method.^61^ Contributions to the MIRC strength of all orbitals are reported in the SI.

**Table 3:** Orbital contributions to the MIRC strength of C_13_Cl_2_ grouped by orbitals type of the *C*_2v_ and *C*_2_ structures. π-in denotes the horizontal π orbitals and π-out denotes the vertical ones. Helical is the total contribution from the three helical π orbitals in Fig. 1

Struct.	Core	Valence σ	π-in	π-out	Helical	Total
*C*_2_	6.1	−0.1	6.1	−5.6	−12.7	−6.2
*C*_2v_	5.9	0.5	3.4	21.7		31.5

Although the *C*_2_ structure is not planar, the orbitals can be grouped into the same kind of orbitals as for the *C*_2v_ structure due to the small deformation of the ring. However, an orbital type consisting of three helical orbitals can also be identified. The orbital types reported in the SI were obtained by projecting the molecular orbitals of the *C*_2_ structure onto the molecular orbitals of the *C*_2v_ structure. Most of the valence orbitals of the *C*_2_ structure consist of more than 50% of a single type. However, the helical orbitals contain significant contributions from both the π-in and the π-out orbitals.

The *C*_2_ structure is antiaromatic, with an MIRC strength of −6.19 nA T^−1^ at the ωB97X level of theory. The MIRC contribution from the helical HOMO is −12.86 nA T^−1^. The MIRC contribution from HOMO-1 (vertical π-out) is −20.10 nA T^−1^ and from HOMO-2 (horizontal π-in) it is −2.52 nA T^−1^. The total contribution of the helical orbitals to the MIRC strength is −12.7 nA T^−1^, which is strongest in the cumulene segment. The π-out contribution to the MIRC strength of the *C*_2_ structure is paratropic (−5.6 nA T^−1^), which can be compared to the diatropic contribution of 21.7 nA T^−1^ from the π-out orbitals of the *C*_2v_ structure. The paratropic π-out contribution increases near the sp^2^ carbon atoms. The σ-orbital contribution to the MIRC strength is diatropic and increases near the sp^2^ carbon atoms. Its average value (sum of core and valence σ contributions) of 6.0 nA T^−1^ is slightly smaller than for the *C*_2v_ structure suggesting that the *C*_2_ structure is somewhat less strained. The net paratropic MIRC of −6.19 nA T^−1^ agrees well with MIRC strength of −6.24 nA T^−1^, which was obtained using Ampèré–Maxwell's law. The MIRC strengths calculated at various levels of theory are reported in Table 4.

**Table 4:** Ring-current strength (*I* in nA T^−1^) for the *C*_2_ and *C*_2v_ structures of C_13_Cl_2_ calculated at the ωB97X, HF-SCF, MP2, CCSD and CASSCF levels using the molecular structure optimized at the ωB97X/def2-TZVP level. The ring-current strength of the *C*_2_ structure was also calculated at the CCSD/def2-TZVP level using the molecular structure optimized at the same level of theory. The def2-TZVP basis sets were used in the MIRC calculations at the ωB97X, HF, and MP2 levels and the cc-pVDZ basis sets were used at the MP2, CCSD and CASSCF levels

Level	*I* (*C*_2_)	*I* (*C*_2v_)
ωB97X^a^	−6.24	31.5
HF-SCF^a^	−4.63	29.5
MP2^a^	−9.18	40.3
MP2^b^	−9.34	40.2
CCSD^b^	−5.67	31.6
CCSD^b^^,^^c^	−6.48	
ωB97X^a^^,^^c^	−8.36	
CASSCF^d^	−3.68	7.22
CASSCF^e^	−3.43	7.22
CASSCF^f^	−3.77	

a The def2-TZVP basis set was used.

b The cc-pVDZ basis set was used.

c The molecular structure was optimized at the CCSD/def2-TZVP level.

d The (12,12) active space was used.

e The (14,14) active space was used.

f The (16,16) active space was used.

### 3.3 The topology of the MIRC

**Fig. 4:**
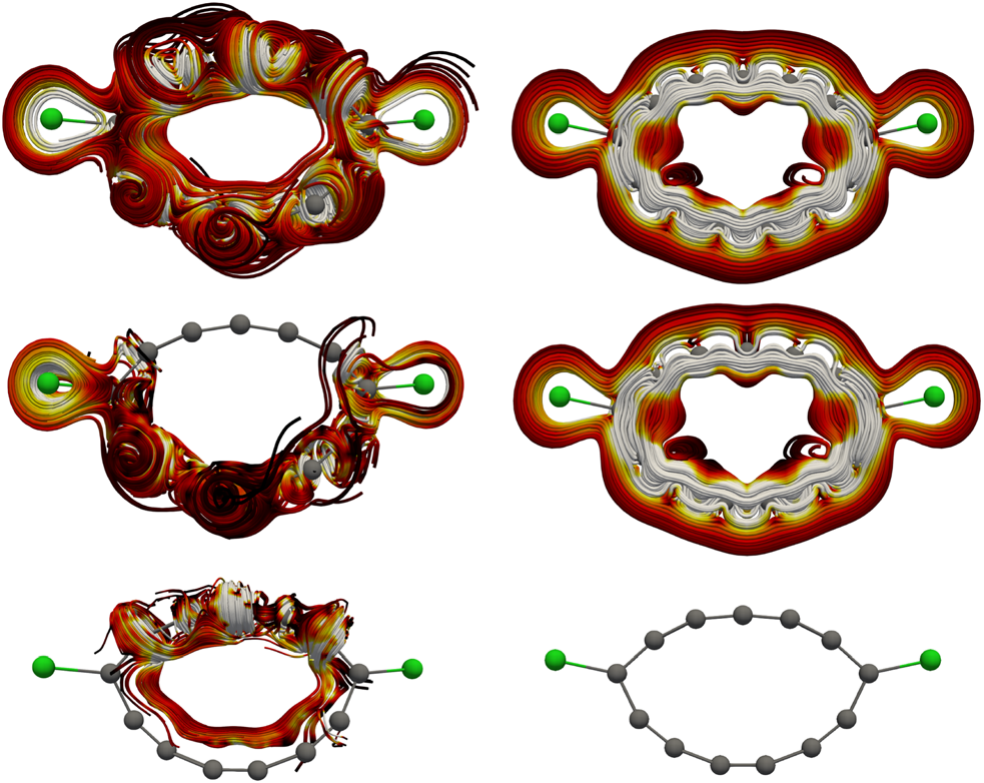
The MICD (top) as well as the diatropic (middle) and paratropic (bottom) MICD contributions of the *C*_2_ (left) and *C*_2v_ (right) structures of C_13_Cl_2_ calculated at the ωB97X level of theory. The white streamlines are strongest. The black ones are weakest with red as intermediate strength.

The MIRC in Fig. 4 shows that the MIRC of the *C*_2_ structure makes vertical loops around the cumulene part of the ring. The diatropic MICD is sustained mainly along the longer segment, whereas the paratropic current flows around the shorter segment and on the inner side of the longer segment. The MICD of the two structures are shown in Fig. 4. Profiles of the MIRC were determined by stepwise integration of the MIRC passing through planes that cut each carbon–carbon bond (see the SI). The same net MIRC strength of about −6.2 nA T^−1^ was obtained for each plane due to charge conservation. The topology of the MIRC is characterized by the twist (*T*_w_) and the writhe (*W*_r_). The MIRC integrations at the C_2_–C_3_ and C_12_–C_13_ bonds reveal localized MICD circulations in the vicinity of these bonds. Strong diatropic and paratropic MIRC strengths pass through integration planes indicating local MICD vortices.

The strongest MICD streamlines have very large *L*_k_ values, which can be seen by inspection. The Cartesian coordinates of the MIRC trajectories used in the topology studies are reported in the SI. The MIRC trajectories can make up to five loops around the cumulene part of the ring, which suggests that the MIRC pathway has an *L*_k_ value of 10. Since one reaches the starting point when following the trajectory around the molecular ring, the *L*_k_ value is most likely even. MIRC trajectories with three, four and five loops are observed, yielding *L*_k_ values of 6, 8, and 10, respectively because each loop increases |*L*_k_| by 2. For example, when *L*_k_ is calculated for ribbons constructed from trajectories making four loops around the cumulene part of the ring, as shown in the SI, one obtains *L*_k_ = 8 consisting of *T*_w_ = −0.02 and *W*_r_ = 8.02. Thus, the MIRC has not a unique *L*_k_ value since its *L*_k_ value depends on the pathway. Calculations on the strongest MIRC trajectories yielded an *L*_k_ value of 6 consisting of *T*_w_ = 0.24 and *W*_r_ = 5.76.

The helical HOMO makes three loops around the molecular framework suggesting that its *L*_k_ value is 6. The topology of the HOMO can also be calculated using the Rappaport–Rzepa approach. The edges of the ribbon can be defined by following the molecular backbone and the largest positive or negative amplitude of the orbital. The topology calculation yields an *L*_k_ value of 6 consisting of *T*_w_ = 5.94 and *W*_r_ = 0.06, which agrees with the *L*_k_ value obtained by visual inspection. The *L*_k_ number must be even when the helical structure extends around the whole molecular ring due to the periodicity. Determining the topology from changes in the phase of the atomic p orbitals is not a reliable approach because the horizontal and vertical p orbitals strongly mix when forming the molecular orbitals.

The calculations indicate that the MICD-derived ribbon and the HOMO-constructed ribbon have the same overall topology around the molecular ring, although their decomposition into *T*_w_ and *W*_r_ differs.

### 3.4 Aromatic nature

The calculated MICD of the *C*_2v_ structure in Fig. 4 shows that it is aromatic sustaining a strong MIRC of 31.5 nA T^−1^ at the ωB97X level. The large MIRC strength of 40.3 nA T^−1^ at the MP2 level cannot be considered a reliable prediction due to the strong electron-correlation effects. The MIRC strength calculated at the CCSD level is 31.6 nA T^−1^, which is in agreement with the MIRC strength calculated at the ωB97X level, while the MIRC strength is weaker at the CASSCF levels, which suggests that dynamic correlation effects are important. The MIRC calculated at the CASSCF level is less reliable than the one obtained at the CCSD level because significant dynamic correlation effects are missing even when we use a very large active space.

Separation of the MICD into diatropic and paratropic contributions shows that the MIRC of the *C*_2v_ structure is almost entirely diatropic flowing smoothly around the planar ring.

The MIRC of the *C*_2_ structure is dominated by its paratropic contribution suggesting that it is antiaromatic. The paratropic contribution to the MIRC passes inside the longer segment of the ring and makes many vertical loops around the cumulene segment (see Fig. 4 and the SI). The MIRC strength of the *C*_2_ structure is −6.24 nA T^−1^ at the ωB97X level, which is in good agreement with the MIRC strength of −5.67 nA T^−1^ calculated at the CCSD level, while at the CASSCF(16,16) level the MIRC strength is only −3.77 nA T^−1^. The MIRC strength is −8.36 nA T^−1^ at the ωB97X level and −6.48 nA T^−1^ at the CCSD level, when using the molecular structure optimized at the CCSD level. The MIRC strengths obtained at different levels of theory are compared in Table 4. The MIRC of the *C*_2_ structure calculated at the ωB97X level using the molecular structure optimized at the CASPT2 level is −6.50 nA T^−1^. The corresponding calculation using the CASSCF molecular structure yields an MIRC strength of −10.03 nA T^−1^ showing that the weak MIRC strength obtained at the CASSCF level is mainly due to the employed electronic structure theory level.

The aromatic nature of the *C*_2v_ structure and the antiaromatic nature of the *C*_2_ structure of C_13_Cl_2_ suggest that their topology has even *L*_k_ and odd *L*_k_, respectively, which is not supported by the topology calculations. The planar *C*_2v_ structure is Hückel aromatic with *L*_k_ = 0. Calculations of the topology of the MIRC of the HOMO and molecular structure of the *C*_2_ structure yielded even *L*_k_ values indicating that the different aromatic nature of the two structures is not due to Hückel and Möbius aromaticity rules, respectively. Calculating the number of orbitals in the aromatic pathway is challenging for C_13_Cl_2_. The *C*_2v_ structure has three occupied frontier orbitals, whose orbital energies are separated from the rest. The orbital energies are reported in the SI. The three occupied frontier orbitals housing six (4*n* + 2) electrons can be the reason for the Hückel aromaticity of the *C*_2v_ structure of C_13_Cl_2_. The HOMO and HOMO-1 orbitals of the C_2_ structure of C_13_Cl_2_ sustain strong paratropic MIRC leading to its antiaromatic nature. It is Hückel (4*n*) antiaromatic when considering the four electrons in these two orbitals.

## 4 Summary and conclusions

We have calculated MICD susceptibility using the GIMIC method and analyzed the MICD of the *C*_2_ and *C*_2v_ molecular structures of C_13_Cl_2_ when the external magnetic field is perpendicular to the molecular ring. The magnetic field induces a strong diatropic MIRC in the planar C_13_Cl_2_ ring of *C*_2v_ symmetry, whereas the C_13_Cl_2_ ring belonging to the *C*_2_ point group sustains a weak paratropic MIRC. The *C*_2v_ structure is aromatic and the *C*_2_ structure can be considered to be weakly antiaromatic.

Rončević *et al.*^1^ investigated the topology of the *C*_2_ structure of C_13_Cl_2_ and introduced the half-Möbius topology concept, which is not supported by the Călugăreanu–White–Fuller theorem. Following the instructions of Rončević *et al.*^1^ when wrapping a paper ribbon around a cylinder yields a Möbius structure with an even *L*_k_ number. The exact *L*_k_ number depends on how one wraps and twists the paper ribbon. Calculations of the topology of the HOMO and the MIRC yield even nonzero *L*_k_ values for them. Since the molecular ring of the *C*_2_ structure is not twisted, it has *L*_k_ = 0. The helical HOMO has an *L*_k_ value of 6. The strongest MIRC streamlines have an *L*_k_ value of 6 consisting of the average *T*_w_ = 0.24 and *W*_r_ = 5.76 values. The paper ribbon has |*L*_k_| = 4 or |*L*_k_| = 8 when one wraps it four times around a cylinder and twists the end by 360° before connected the ends to a closed ribbon. Thus, a similar topology is obtained for the paper ribbon as for the HOMO and the strongest streamlines of the MIRC when omitting the double twist of the ribbon. Topology calculations of the MICD also suggest that the *T*_w_ contribution to *L*_k_ is very small. Visual inspection of the MICD showed that the MIRC of the *C*_2_ structure has a complicated topology, where the MIRC makes several loops around the cumulene part of the ring. Some MIRC streamlines make four or even five loops around the cumulene part of the ring yielding *L*_k_ = 8 with the average *T*_w_ = −0.02 and *W*_r_ = 8.02 values when the MIRC makes four loops. Thus, the topology of the MIRC is not unique. The *L*_k_ value depends on the pathway since every loop the MIRC makes around the cumulene part of the ring increases |*L*_k_| by 2. Every MIRC trajectory has its own *T*_w_ and *W*_r_ value because *T*_w_ and *W*_r_ depend on the details of the MIRC pathway, whereas many MIRC trajectories have the same *L*_k_ value and thus the same topology. The *C*_2v_ structure is Hückel aromatic and the *C*_2_ structure of C_13_Cl_2_ is Hückel antiaromatic because they have even *L*_k_ values and sustain net diatropic and net paratropic MIRC, respectively.

The present study shows that there is no need to introduce the half-Möbius topology concept to understand the electronic structure and the magnetic response of the *C*_2_ molecular structure of C_13_Cl_2_.

## Author contributions

The research topic was suggested by DS, who wrote the first version of the manuscript. QW, RTN, RRV, SB, JG, and DS performed calculations. RTN constructed MIRC ribbons and determined their topology. RTN and QW performed MICD calculations and made pictures and videos. All authors have contributed to the final version of the article.

## Conflicts of interest

There are no conflicts of interest to declare.

## Supplementary Material

SC-017-D6SC03398A-s001

SC-017-D6SC03398A-s002

SC-017-D6SC03398A-s003

## Data Availability

The data supporting this article have been included as part of the supplementary information (SI). DFT, MP2 and CCSD calculations were performed with Turbomole version 7.9. The Turbomole web page is https://www.turbomole.org/. CCSD and CASSCF computations of the nuclear magnetic shieldings were performed with a development version of CFOUR. The CFOUR web page is https://https://cfour.uni-mainz.de/cfour/. GIMIC, version 2.0 can be freely downloaded from https://github.com/qmcurrents/gimic and https://zenodo.org/record/8180435. ParaView and VESTA are free software that can be downloaded from https://www.paraview.org/ and https://jp-minerals.org/vesta/, respectively. The source code of BAGEL can be freely downloaded from https://github.com/nubakery/bagel. The source code of GAMESS-US can be freely downloaded from https://www.msg.chem.iastate.edu/gamess/. Supplementary information: pictures of molecular orbitals, MICD pictures and videos, orbital contributions to the MIRC strengths, the coefficient of the dominating configuration, isotropic magnetic shielding constants, excitation energies calculated at the XMS-CAPT2 level, bond lengths, and the Cartesian coordinates of the optimized molecular structures. See DOI: https://doi.org/10.1039/d6sc03398a.
