# Supplementary material for: The topology of the magnetically induced ring current of C13Cl2
Source: Chem Sci. 2026 Jun 27;17(32):15400–8. doi: 10.1039/d6sc03398a (PMC13334967; doi:10.1039/d6sc03398a)
Supplement: Supplementary file 1 [file SC-017-D6SC03398A-s003.pdf]

Supplementary Information (SI) for Chemical Science.
This journal is © The Royal Society of Chemistry 2026

Electronic Supplementary Information

The Topology of the Magnetically Induced Ring Current of $C_{13}Cl_2$

Molecular orbitals

Table S1: The molecular orbitals of the C_2 structure of $C_{13}Cl_2$.

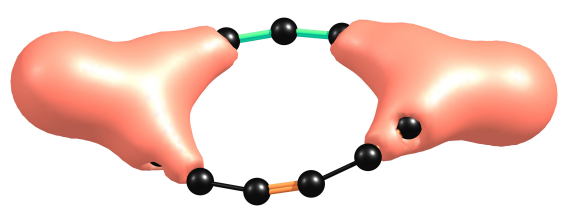
24(a, σ)

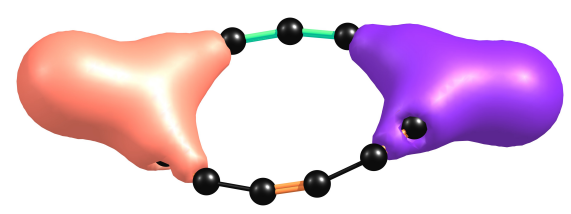
25(b, σ)

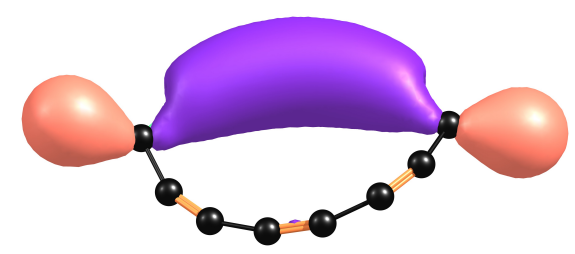
26(a, σ)

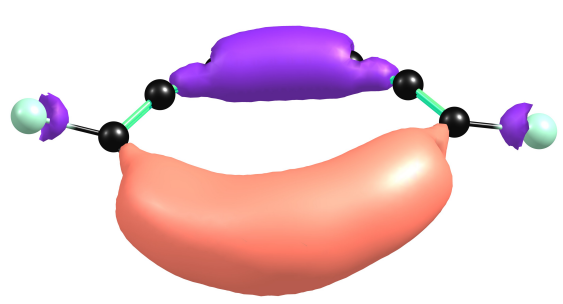
27(a, σ)

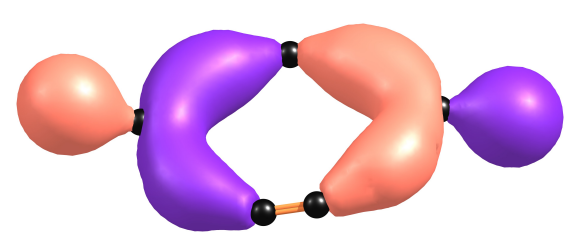
28(b, σ)

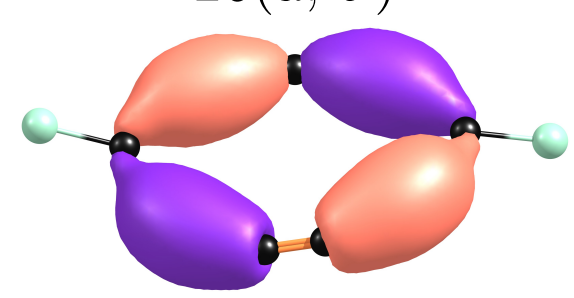
29(b, σ)

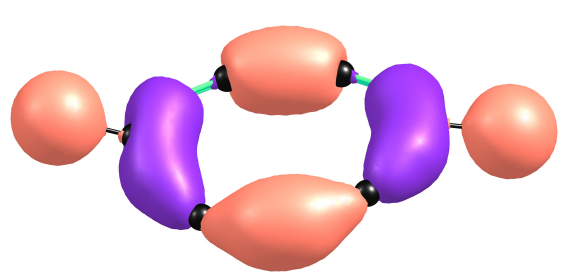
30(a, σ)

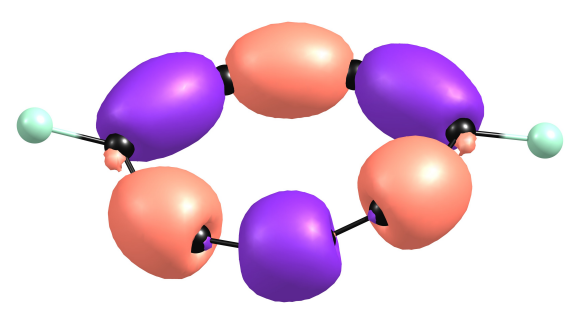
31(a, σ)

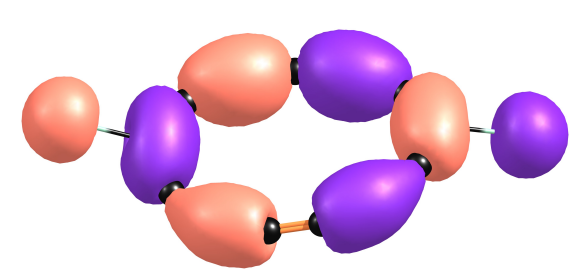
32(a, σ)

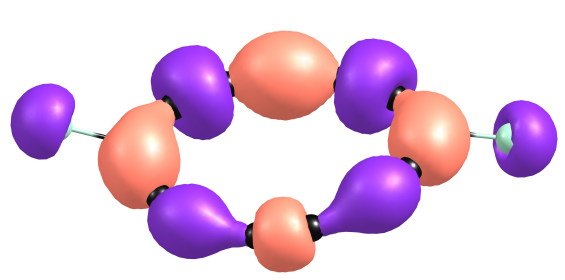
33(a, σ)

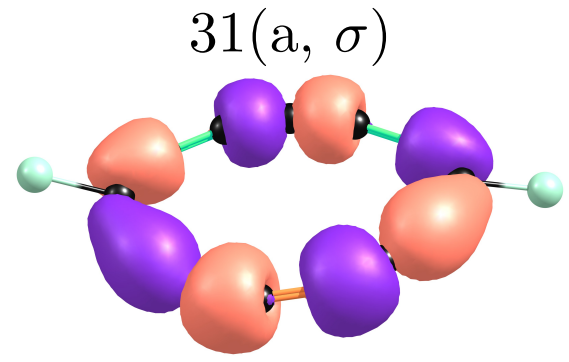
34(b, σ)

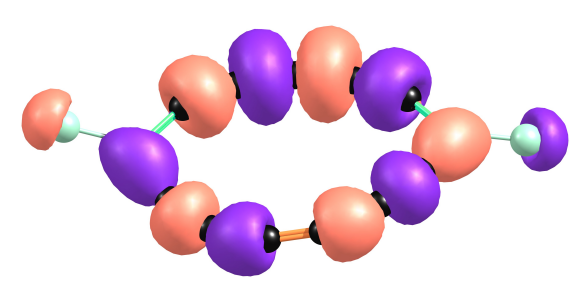
35(b, σ)

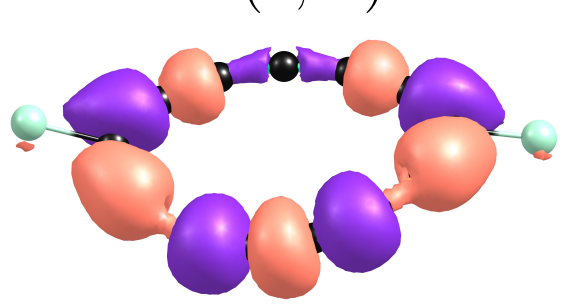
36(a, σ)

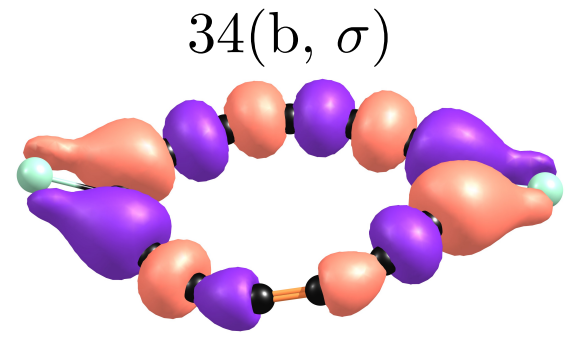
37(b, σ)

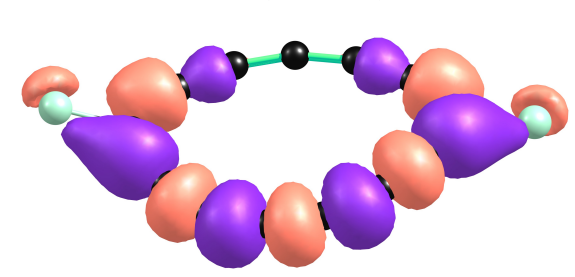
38(a, σ)

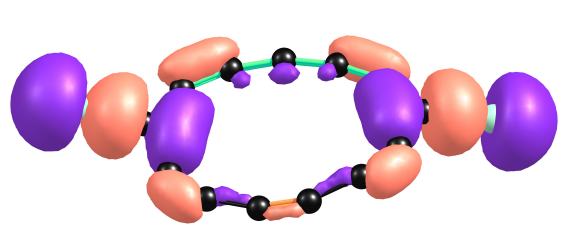
39(a, π-in)

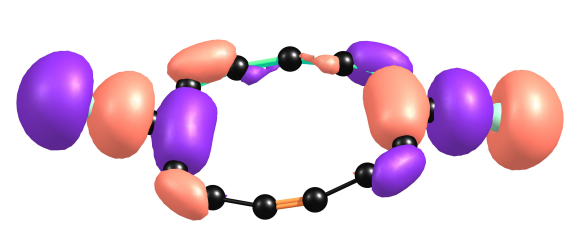
40(b, π-in)

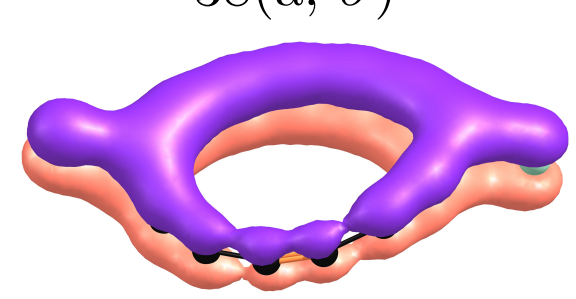
41(b, π-out)

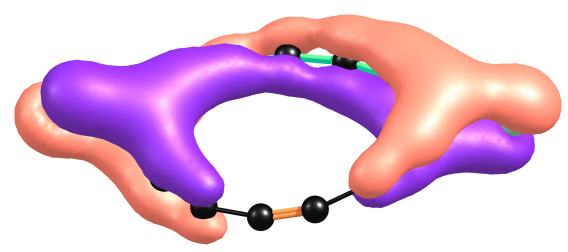

42(a, π-out)

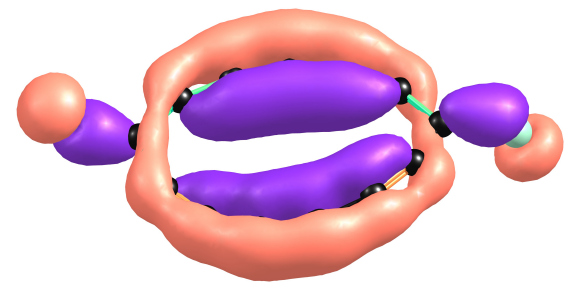

43(a, π-in)

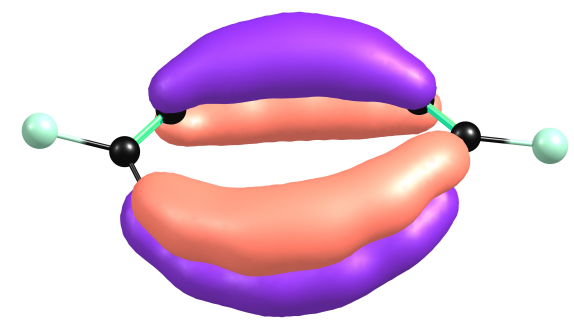

44(b, π-out)

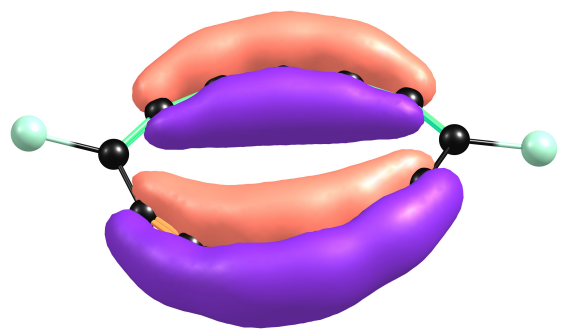

45(a, π-in)

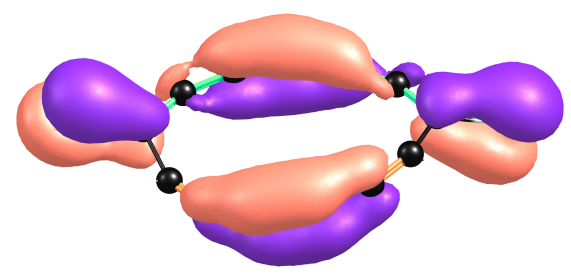

46(b, π-out)

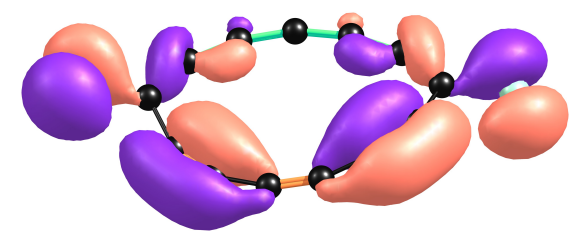

47(b, π-in)

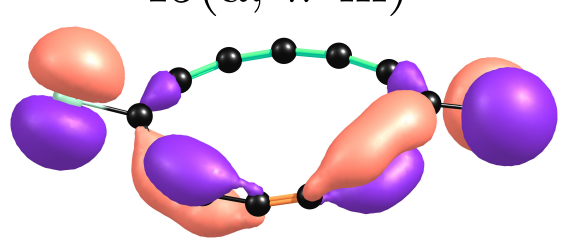

48(a, π-out)

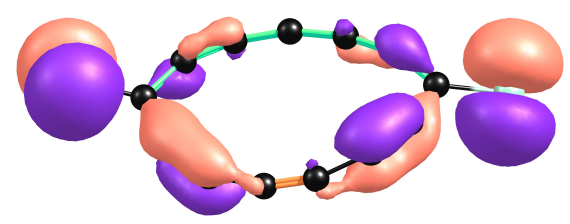

49(a, π-out)

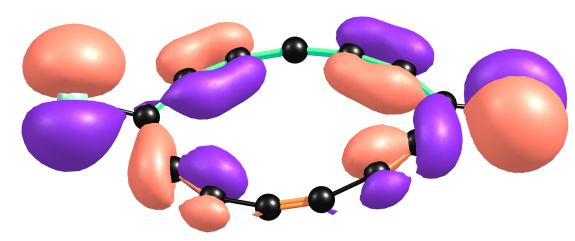

50(b, π-in)

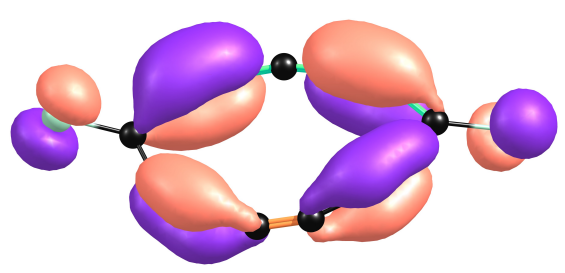

51(a, π-out)

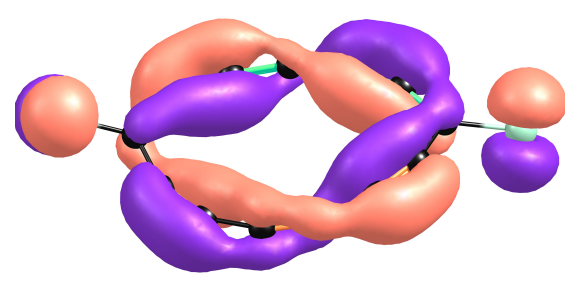

52(b, helix)

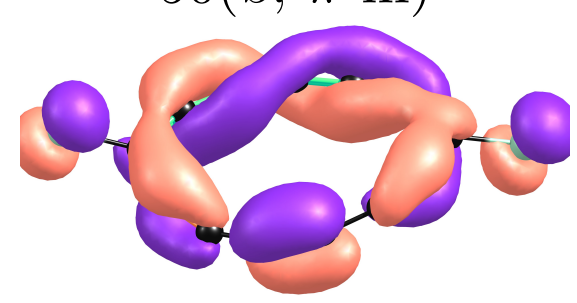

53(b, helix)

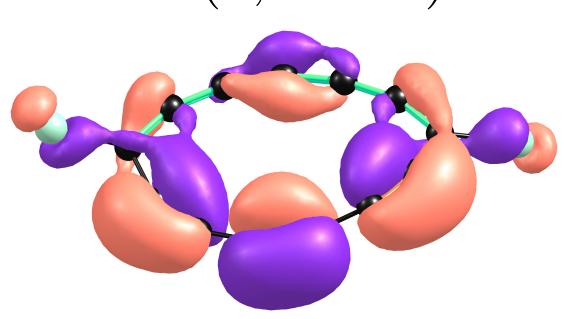

54(a, π-in)

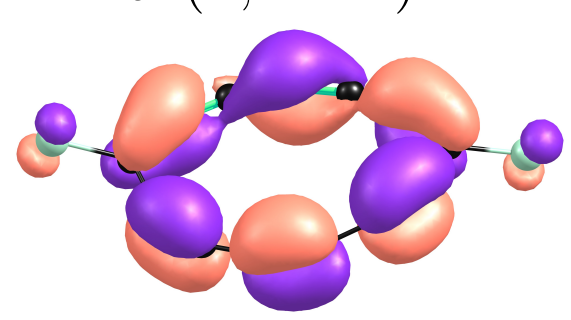

55(b, π-out)

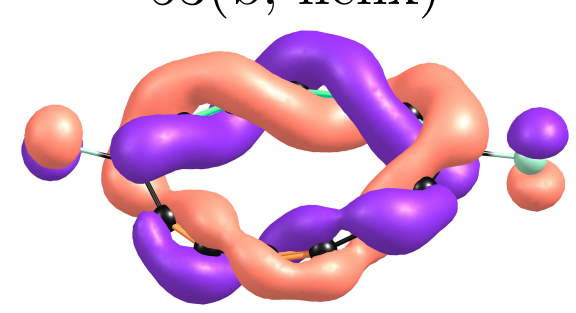

56(a, helix)

Table S2: The molecular orbitals of the $C_{2\mathrm{v}}$ structure of $C_{13}Cl_2$.

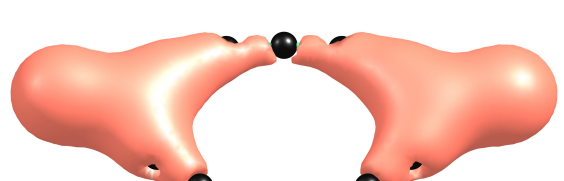

24(a, σ)

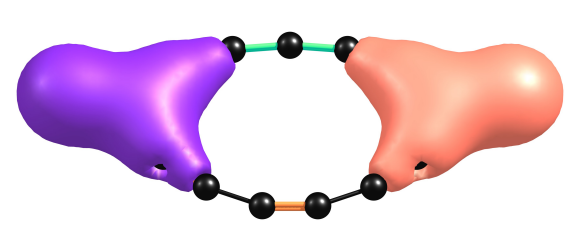

25(b, σ)

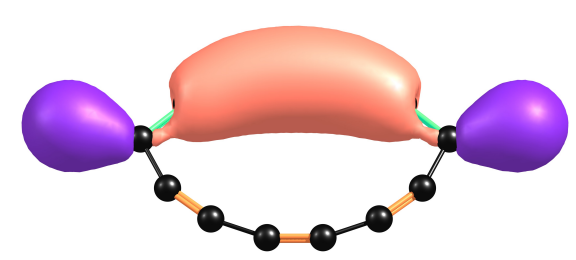

26(a, σ)

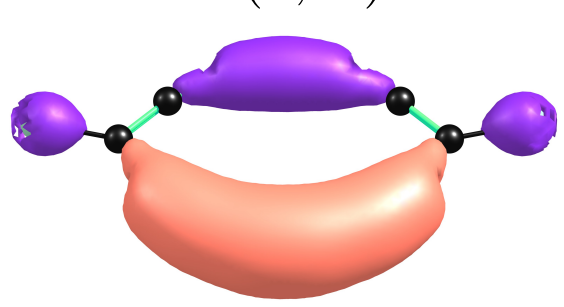

27(a, σ)

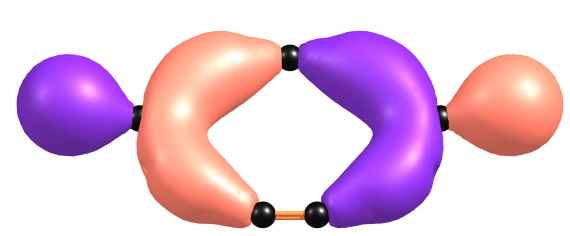

28(b, σ)

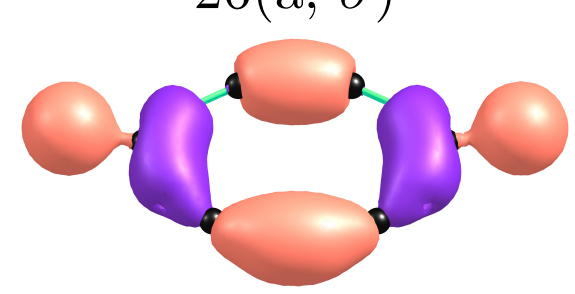

29(b, σ)

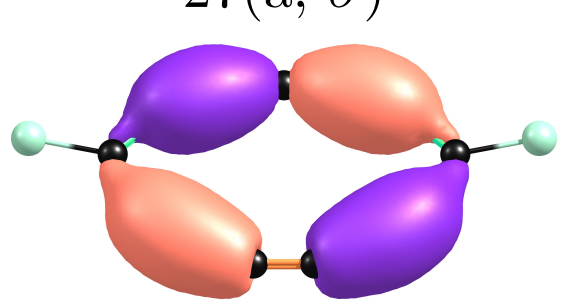

30(a, σ)

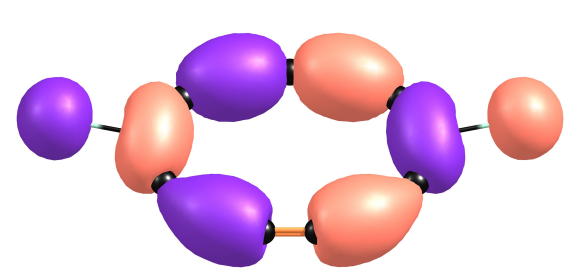

31(a, σ)

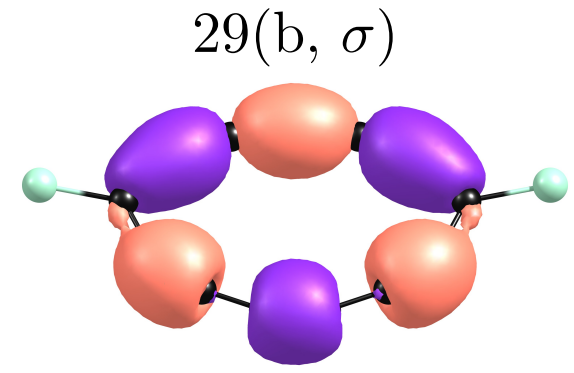

32(a, σ)

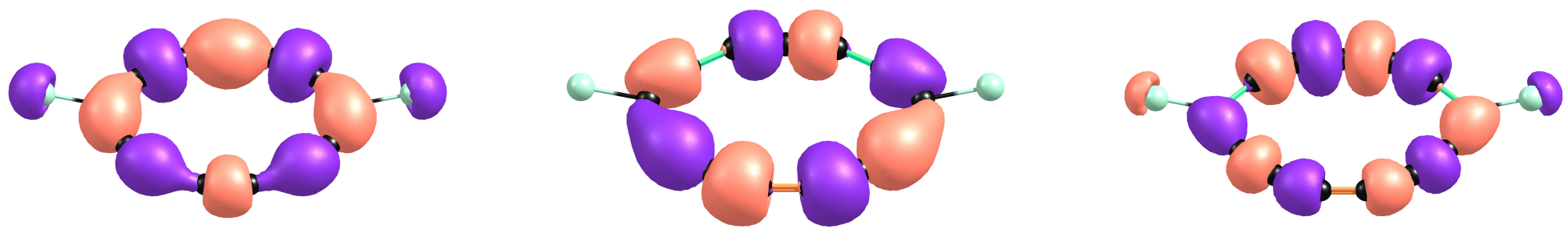

33(a, σ) 34(b, σ) 35(b, σ)

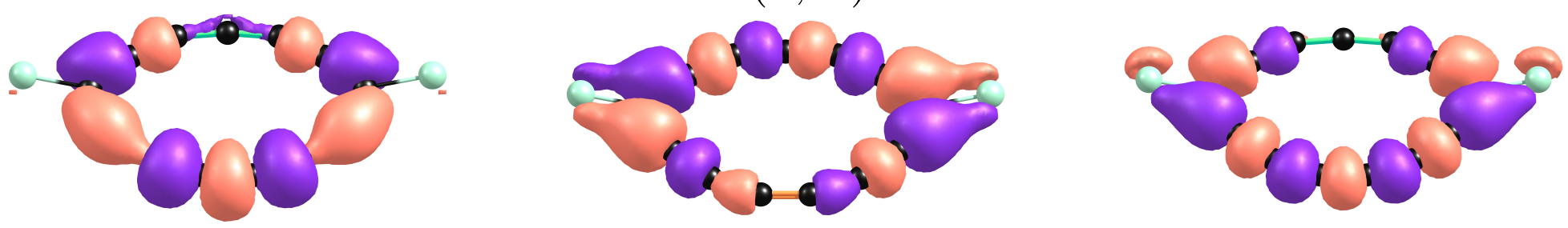

36(a, σ) 37(b, σ) 38(a, σ)

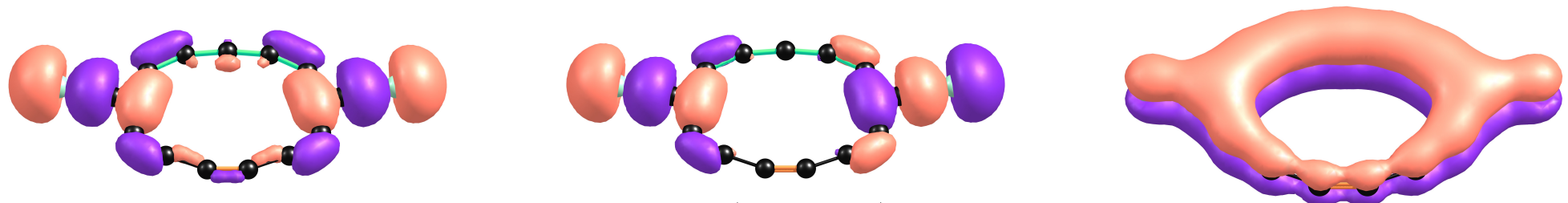

39(a, π-in) 40(b, π-in) 41(b, π-out)

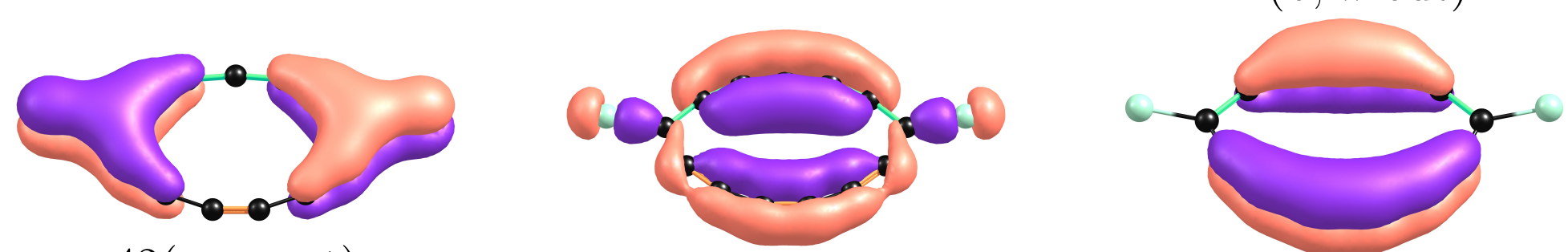

42(a, π-out) 43(a, π-in) 44(b, π-out)

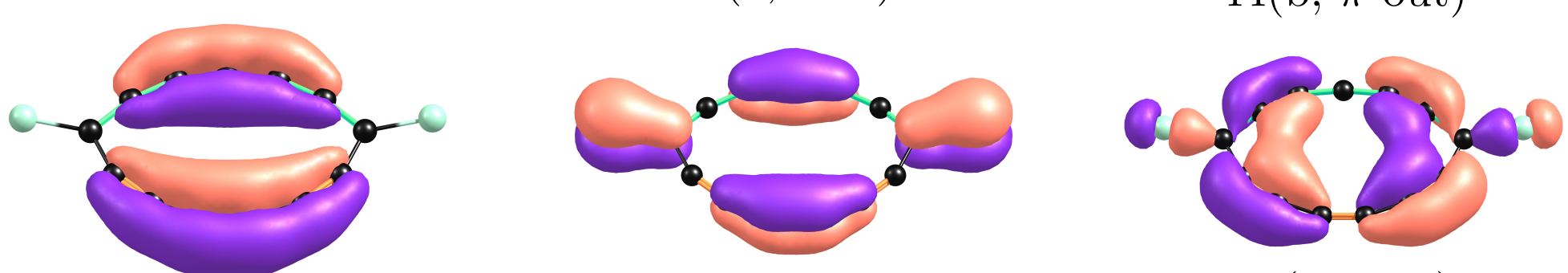

45(a, π-in) 46(b, π-out) 47(b, π-in)

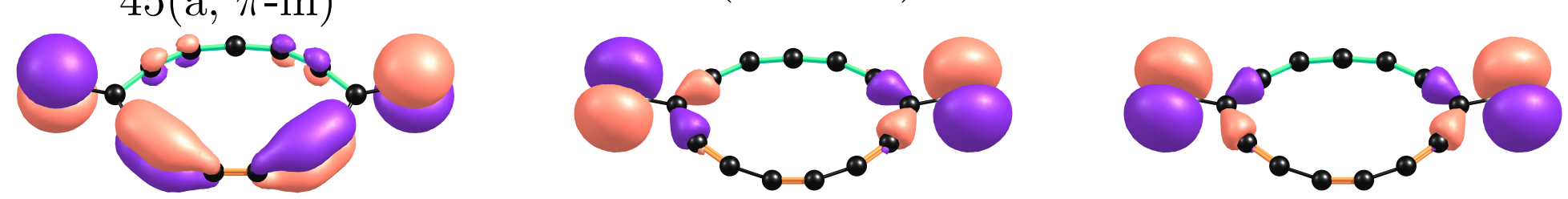

48(a, π-out) 49(a, π-in) 50(b, π-in)

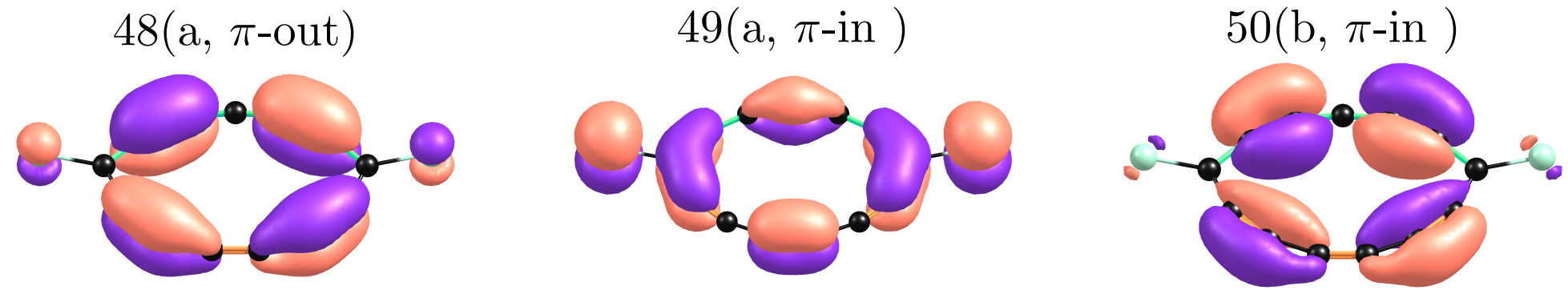

51(a, π-out) 52(b, π-out) 53(b, π-in)

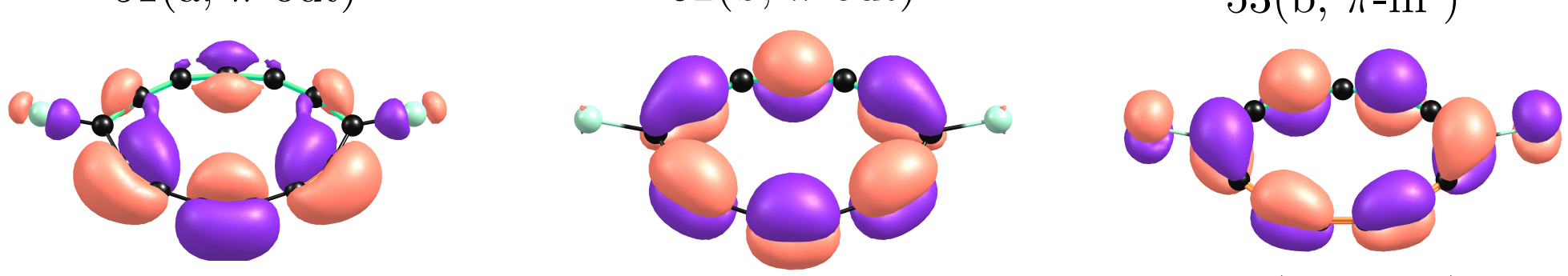

54(a, π-in) 55(b, π-out) 56(a, π-out)

Table S3: The two dominating contributions of the molecular orbitals of the C_{2v} structure to the occupied frontier orbitals of the C_2 structure of $C_{13}Cl_2$.

C_2 MO	C_{2v} MO 1	%	C_{2v} MO 2	%
39(π-in)	39(π-in)	64.76	48(π-in)	4.38
40(π-in)	40(π-in)	62.49	52(π-out)	5.71
41(π-out)	41(π-out)	78.34	28(π-in)	3.33
42(π-out)	42(π-out)	61.56	43(π-in)	10.81
43(π-in)	43(π-in)	73.19	42(π-out)	6.83
44(π-out)	44(π-out)	83.85	9(core)	1.84
45(π-in)	45(π-in)	89.40	108(π-in)	0.79
46(π-out)	46(π-out)	50.64	52(π-out)	7.24
47(π-in)	47(π-in)	47.66	49(π-in)	21.43
48(π-out)	50(π-in)	27.85	48(π-out)	20.04
49(π-out)	50(π-in)	26.02	48(π-out)	11.26
50(π-in)	49(π-in)	34.31	47(π-in)	22.75
51(π-out)	51(π-out)	70.33	50(π-in)	2.01
52(helix)	53(π-in)	56.57	52(π-out)	11.14
53(helix)	52(π-out)	30.17	53(π-in)	26.79
54(π-in)	54(π-in)	79.98	56(π-out)	5.10
55(π-out)	55(π-out)	73.01	11(core)	2.25
56(helix)	56(π-out)	36.79	57(π-in)	36.02

Orbital contributions to the MIRC strengths of $C_{13}Cl_2$

Table S4: The average orbital contributions to the MIRC strength ($\langle I_i \rangle$ in nA/T) of $C_{13}Cl_2$. Orbital energies (ϵ in eV), orbital types and irreducible representations are also given.

	C_{2v}				C_2			
№	Irrep	ϵ	Type	$\langle I_i \rangle$	Irrep	ϵ	Type	$\langle I_i \rangle$
1-23	core		σ	5.93	core		σ	6.06
24	$12a_1$	-28.847	σ	1.49	a	-28.808	σ	1.14
25	$11b_1$	-28.810	σ	2.01	b	-28.782	σ	1.78
26	$13a_1$	-27.264	σ	2.00	a	-27.093	σ	2.46
27	$14a_1$	-26.794	σ	2.55	a	-26.694	σ	2.63
28	$12b_1$	-26.607	σ	0.82	b	-26.390	σ	1.03
29	$15a_1$	-25.601	σ	1.88	b	-25.515	σ	1.36
30	$13b_1$	-25.576	σ	1.22	a	-25.350	σ	1.66
31	$14b_1$	-23.901	σ	1.48	a	-23.895	σ	0.09
32	$16a_1$	-23.792	σ	-0.13	b	-23.588	σ	0.88
33	$17a_1$	-22.092	σ	-0.33	a	-21.893	σ	-0.27
34	$15b_1$	-21.429	σ	-0.38	b	-21.319	σ	-0.32
35	$16b_1$	-20.118	σ	-2.43	b	-19.943	σ	-2.56
36	$18a_1$	-19.542	σ	-2.49	a	-19.523	σ	-2.16
37	$17b_1$	-18.354	σ	-3.36	b	-18.430	σ	-3.58
38	$19a_1$	-18.263	σ	-3.82	a	-18.245	σ	-4.23
39	$20a_1$	-16.898	π-in	1.25	a	-16.788	π-in	1.25
40	$18b_1$	-16.854	π-in	0.90	b	-16.745	π-in	0.92
41	$2b_2$	-15.487	π-out	3.41	b	-15.313	π-out	3.24
42	$2a_2$	-15.081	π-out	3.04	a	-15.044	π-out	2.95
43	$21a_1$	-14.427	π-in	2.56	a	-14.138	π-in	2.24
44	$3b_2$	-14.233	π-out	3.69	b	-14.085	π-out	3.49
45	$22a_1$	-13.814	π-in	3.30	a	-13.848	π-in	3.32
46	$4b_2$	-13.506	π-out	2.15	b	-13.475	π-out	2.00
47	$19b_1$	-12.574	π-in	1.42	b	-12.524	π-in	0.89
48	$3a_2$	-12.457	π-out	1.08	a	-12.377	π-out	0.42
49	$20b_1$	-12.323	π-in	-0.38	a	-12.343	π-out	0.29
50	$23a_1$	-12.308	π-in	-0.44	b	-12.313	π-in	0.02
51	$4a_2$	-12.055	π-out	2.88	a	-11.828	π-out	2.15
52	$5b_2$	-11.248	π-out	1.88	b	-11.352	helix	-0.34
53	$21b_1$	-10.876	π-in	-2.32	b	-10.706	helix	0.47
54	$24a_1$	-9.551	π-in	-2.92	a	-9.921	π-in	-2.52
55	$6b_2$	-8.519	π-out	1.49	b	-8.765	π-out	-20.10
56	$5a_2$	-7.811	π-out	2.04	a	-8.263	helix	-12.86
core				5.93				6.06
σ				0.50				-0.09
π-in				3.37				6.13

Continued on next page

	C_{2v}				C_2			
№	Irrep	ϵ	Type	$\langle I_i \rangle$	Irrep	ϵ	Type	$\langle I_i \rangle$
π-out				21.66				-5.56
helix								-12.73
Total				31.45				-6.19

The MIRC of the C_2 structure of $C_{13}Cl_2$

The Cartesian coordinates of a few MIRC trajectories are given in the trajectory.zip file. A typical MIRC trajectory and the MIRC are shown in Figure S1. The MIRC of the C_2 structure of $C_{13}Cl_2$ and its diatropic and paratropic contributions are shown in Figure S2. The profile of the MIRC of the C_2 structure of $C_{13}Cl_2$ passing through various integration planes is shown in Figure S3. The MIRC of the C_{2v} structure of $C_{13}Cl_2$ and its diatropic and paratropic contributions are shown in Figure S4. The MIRC profile of the C_{2v} structure of $C_{13}Cl_2$ calculated at the ωB97X and MP2 levels are compared in Figure S5. The diatropic and paratropic contributions to the MIRC as well as the MIRC profile calculated for the C_2 and C_{2v} structures of $C_{13}Cl_2$ at different levels of theory are shown in Tables S5 and S6, respectively.

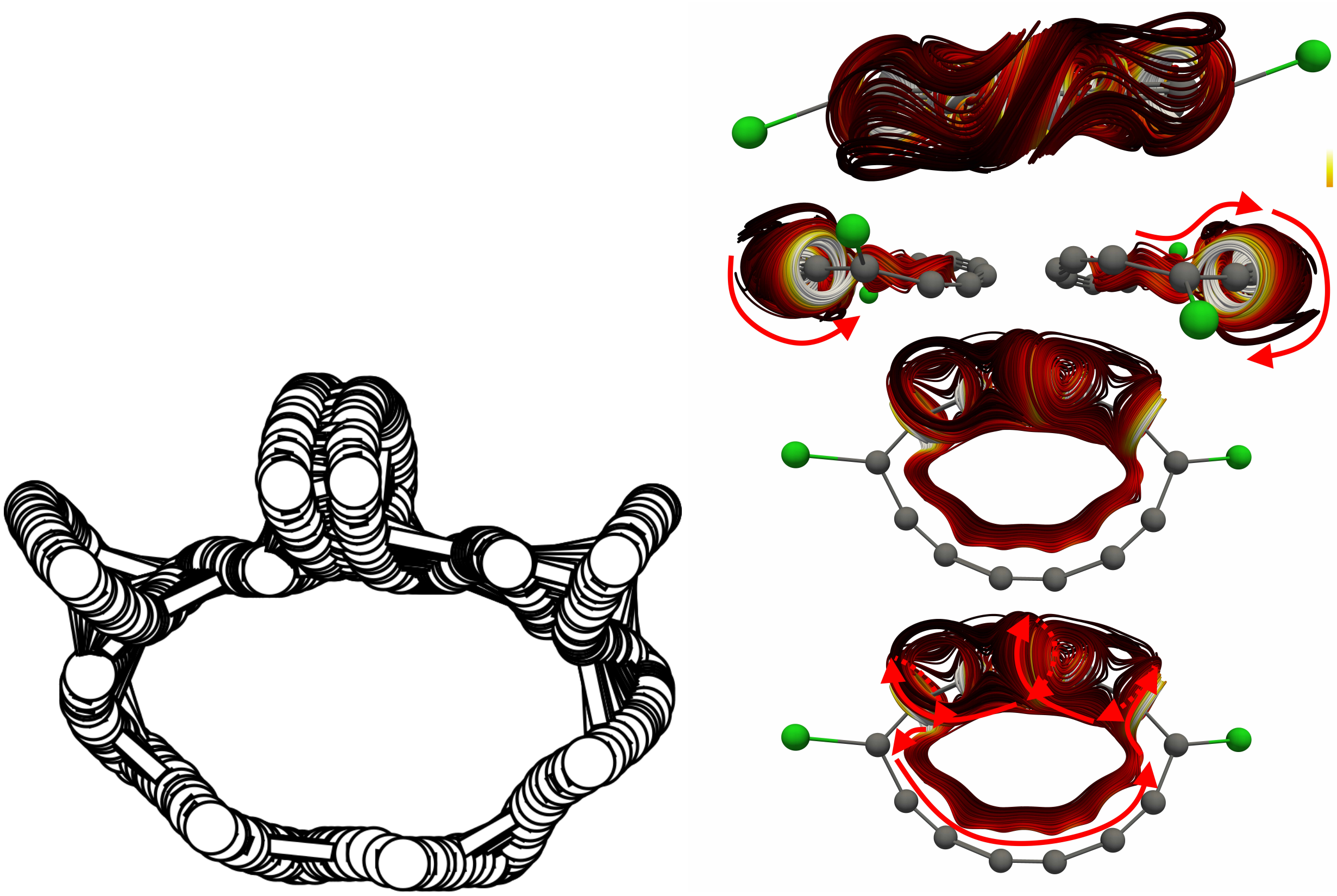

Figure S1: A MIRC trajectory of the C_2 structure of $C_{13}Cl_2$ (left). The MIRC of the C_2 structure of $C_{13}Cl_2$ (right) showing the MIRC loops around the shorter segment of the ring.

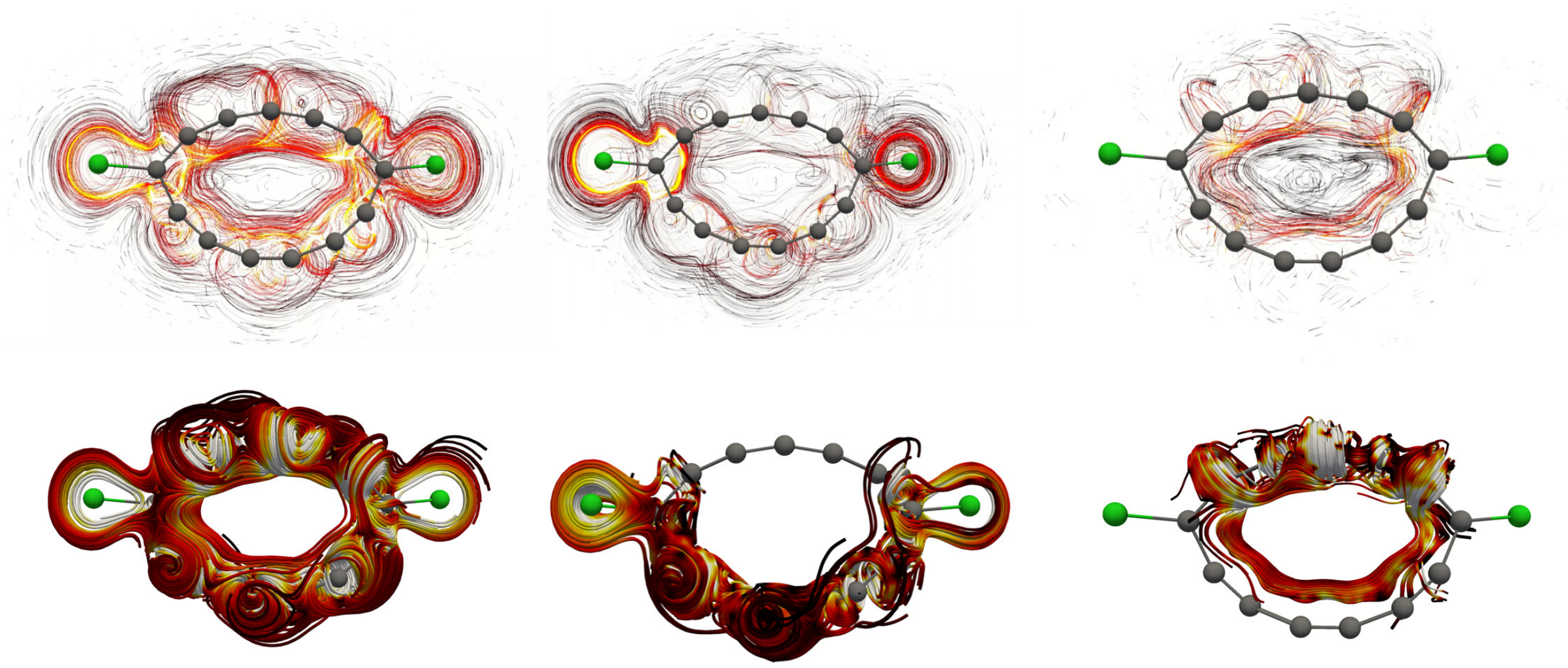

Figure S2: The MICD of the C_2 structure of $C_{13}Cl_2$ (left) and its diatropic (middle) and paratropic (right) contributions calculated at the ωB97X level.

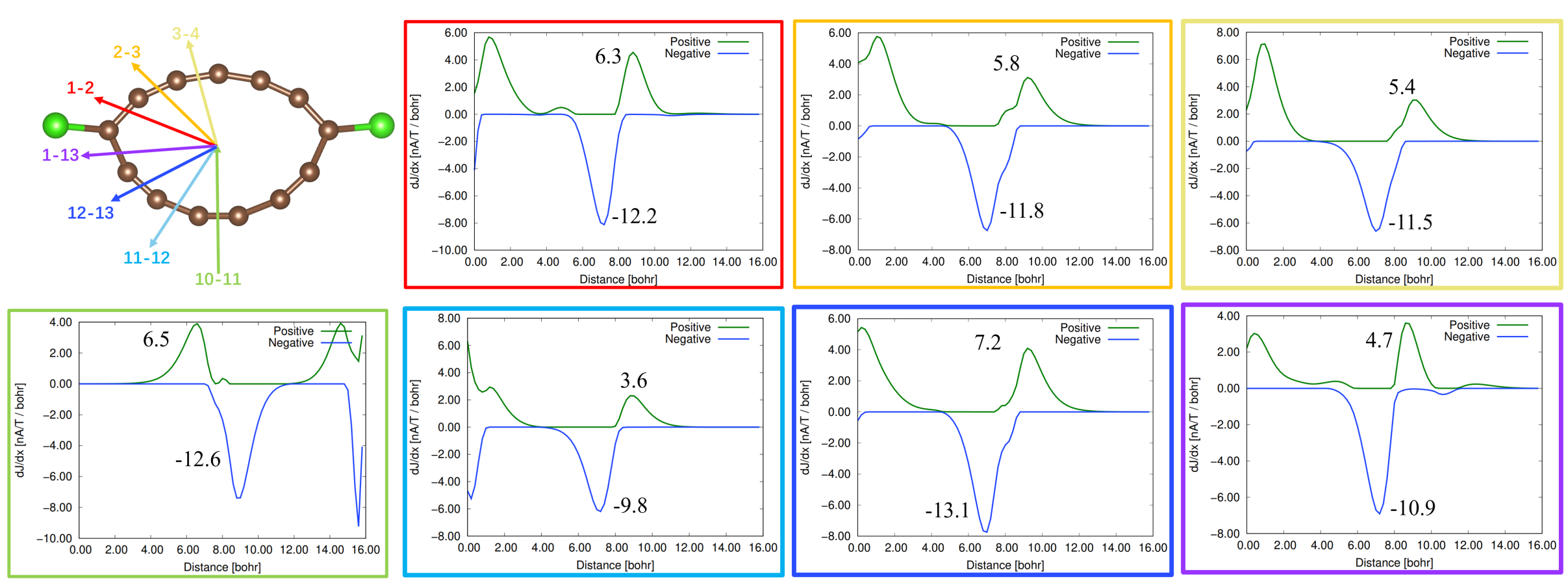

Figure S3: MIRC profiles of the C_2 structure of $C_{13}Cl_2$. The integration plane passes through various chemical bonds. The calculations are performed at the ωB97X level.

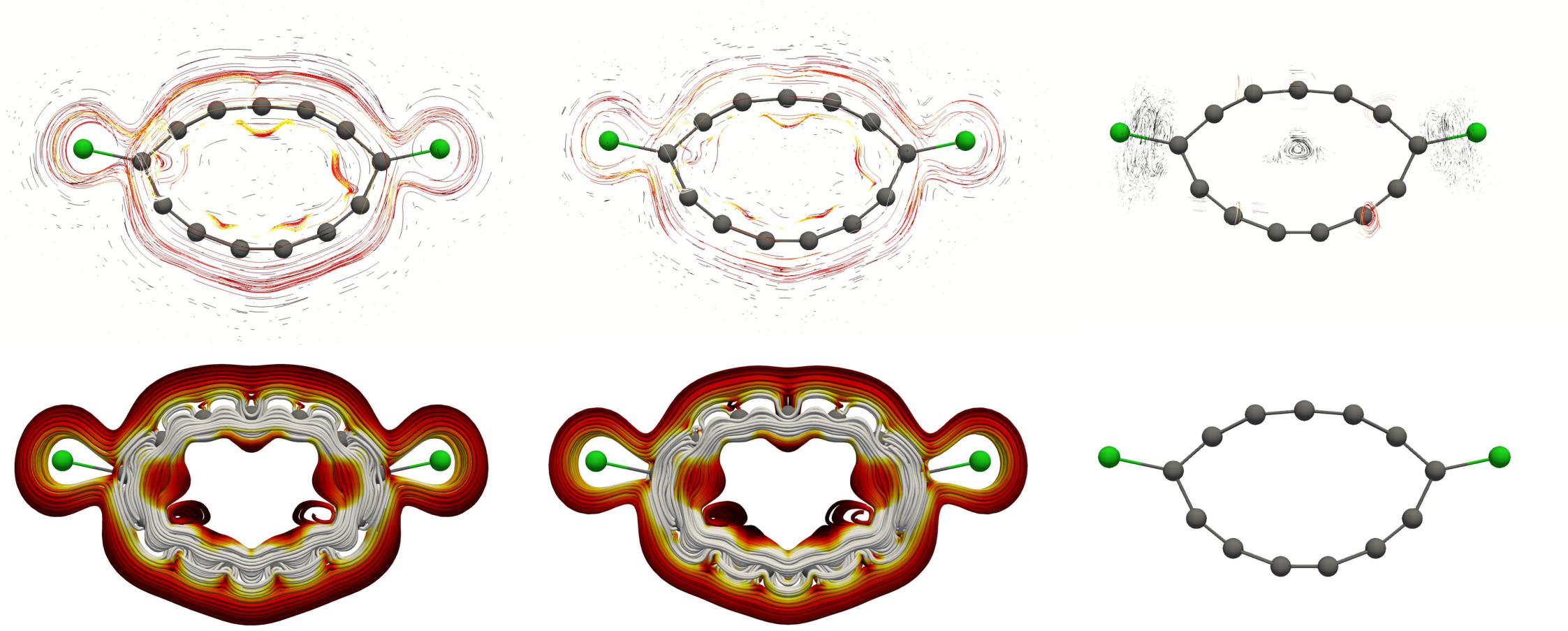

Figure S4: The MIRC of the C_{2v} structure of $C_{13}Cl_2$ (left) and its diatropic (middle) and paratropic (right) contributions calculated at the ωB97X level.

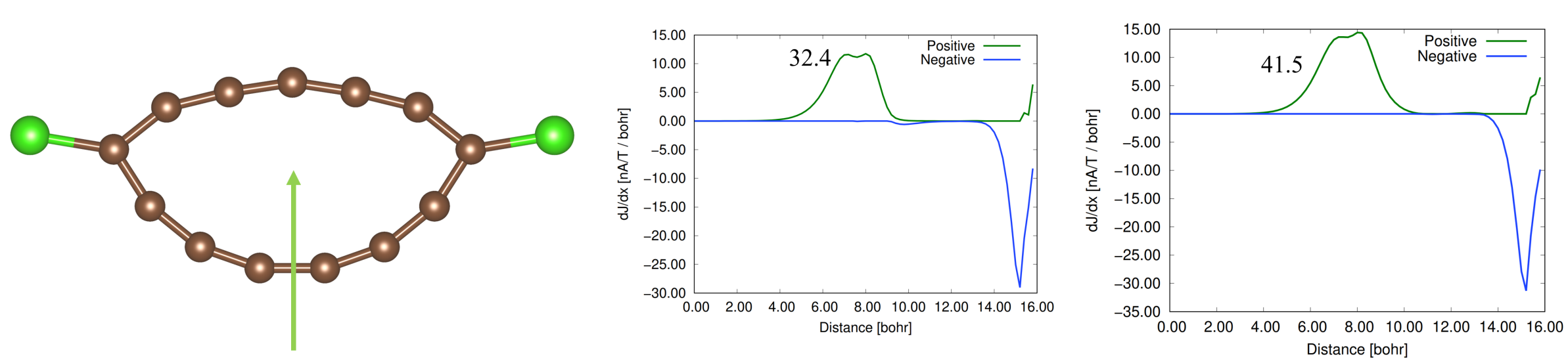

Figure S5: The MIRC profile of the C_{2v} structure of $C_{13}Cl_2$ calculated at ωB97X level (middle) and the MP2 level (right).

Table S5: The diatropic (blue) and paratropic (red) contributions to the MIRC as well as the MIRC profile calculated for the C_2 structure of $C_{13}Cl_2$ at different levels of theory.

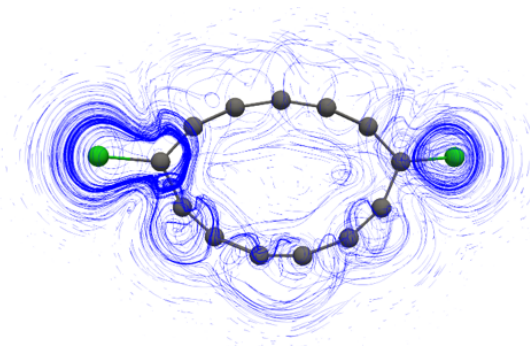
ωB97X – dia

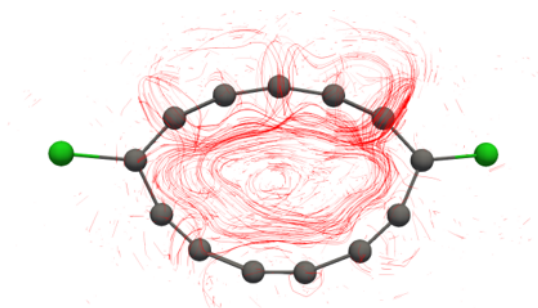
ωB97X – para

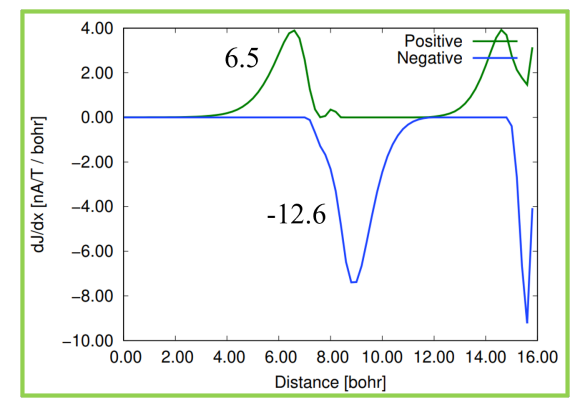

ωB97X – integral

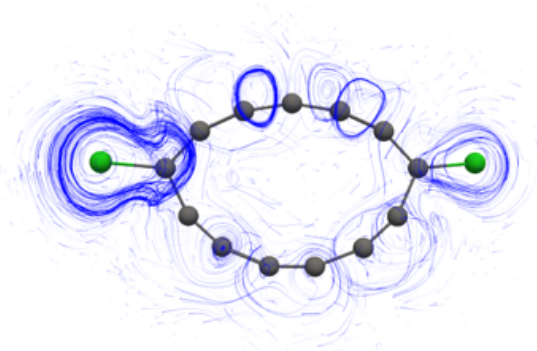
MP2 – dia

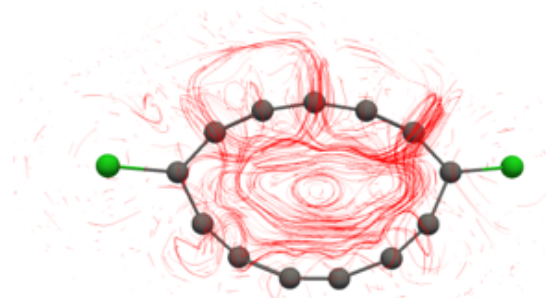
MP2 – para

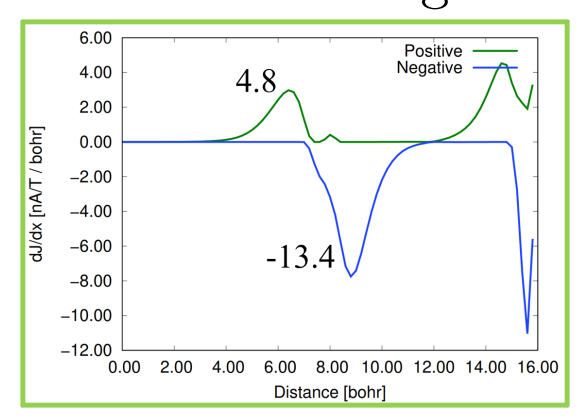

MP2 – integral

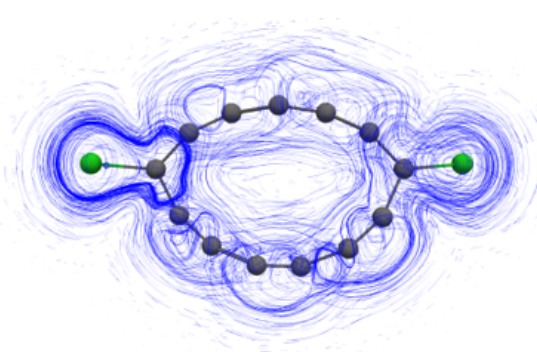
CCSD – dia

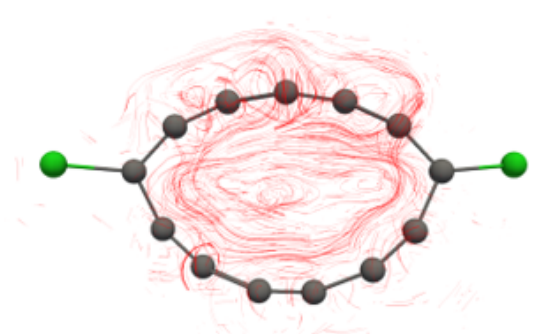
CCSD – para

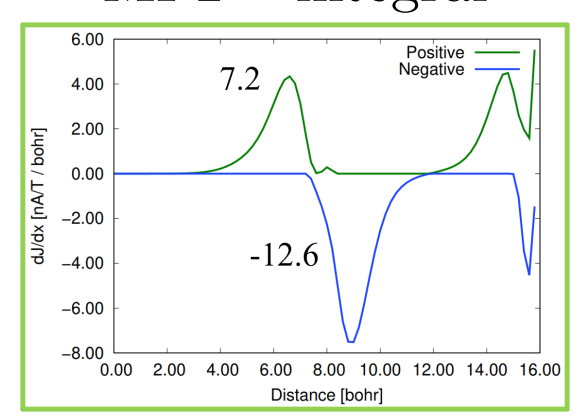

CCSD – integral

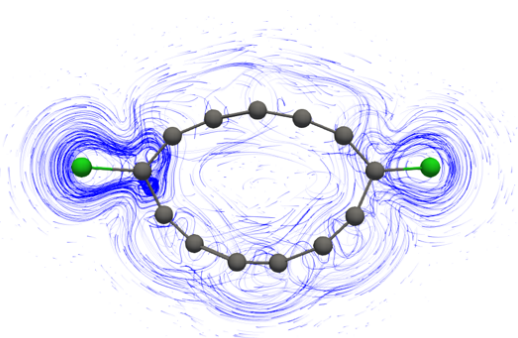
CCSD OPT – dia

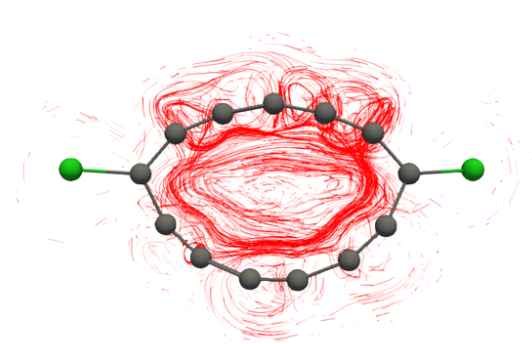
CCSD OPT – para

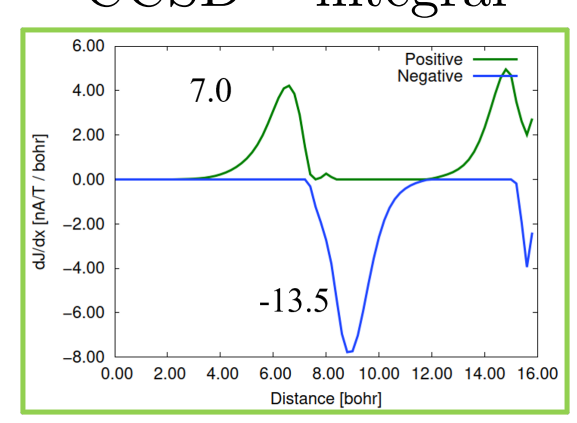

CCSD OPT – integral

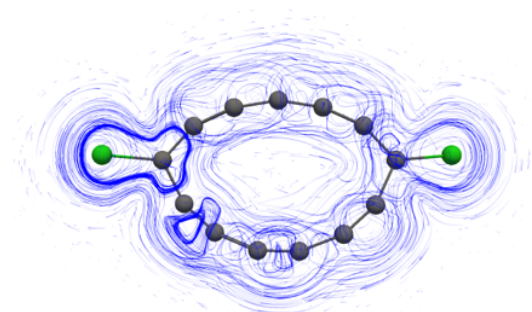
CAS(12 12) – dia

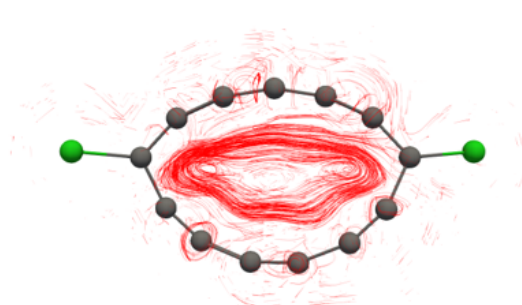
CAS(12 12) – para

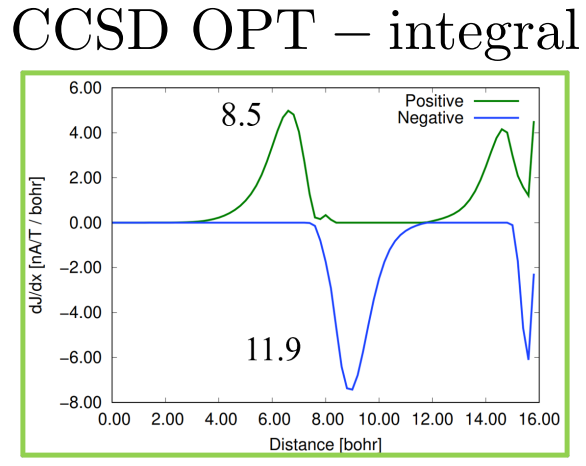

CAS(12 12) – integral

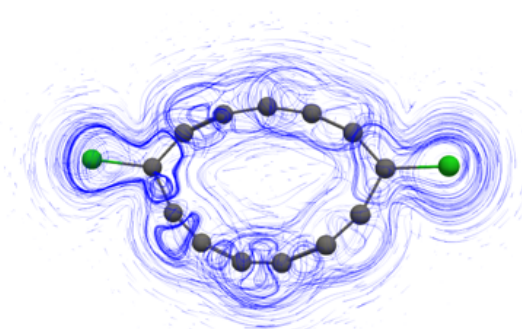
CAS(14 14) – dia

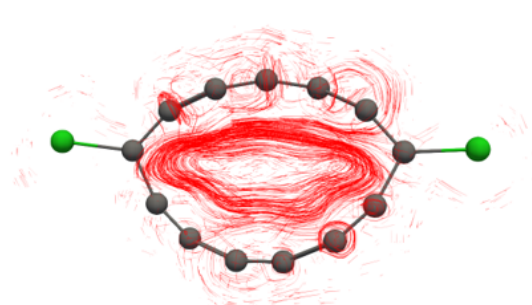
CAS(14 14) – para

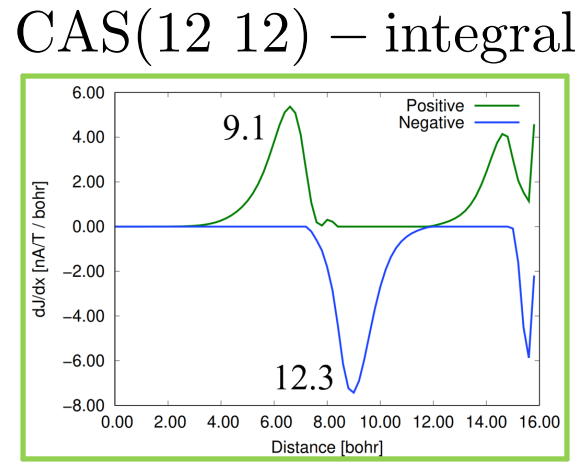

CAS(14 14) – integral

Table S6: The diatropic (blue) and paratropic (red) contributions to the MIRC as well as the MIRC profile calculated for the C_{2v} structure of $C_{13}Cl_2$ at different levels of theory.

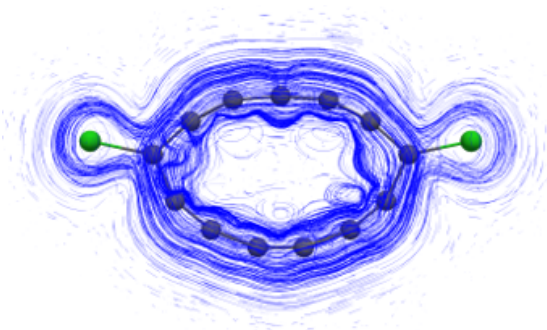

ωB97X – dia

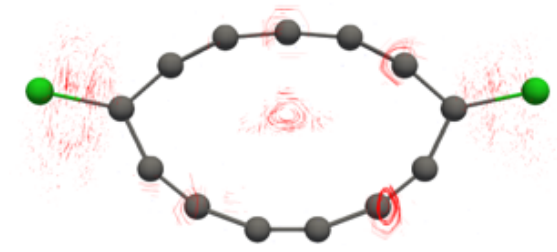

ωB97X – para

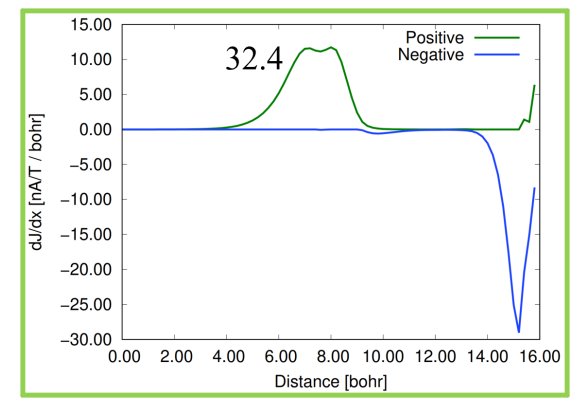

ωB97X – integral

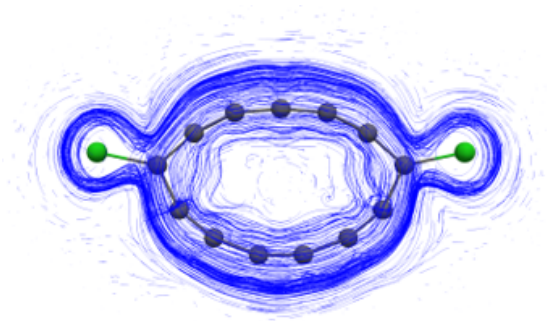

MP2 – dia

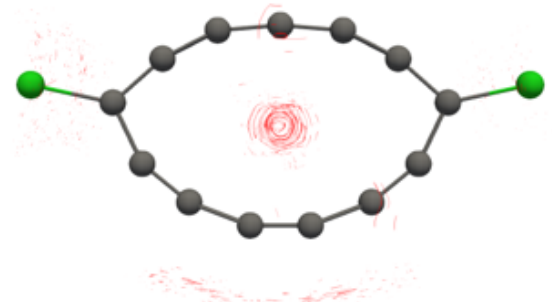

MP2 – para

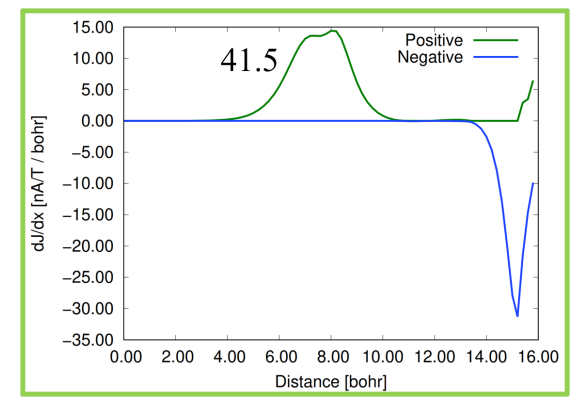

MP2 – integral

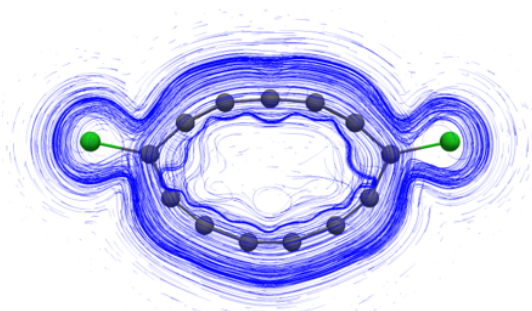

CCSD – dia

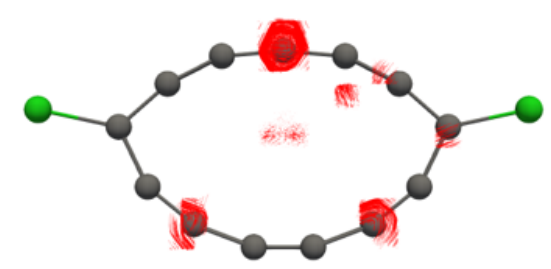

CCSD – para

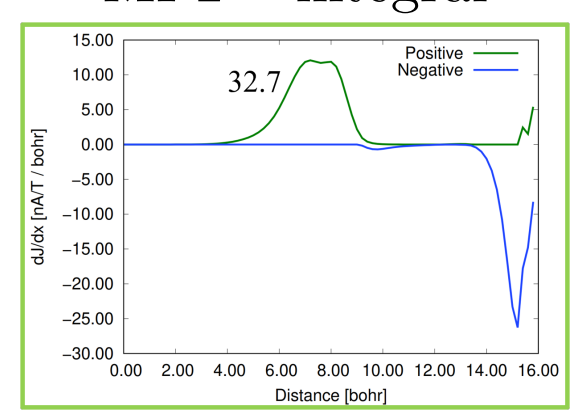

CCSD – integral

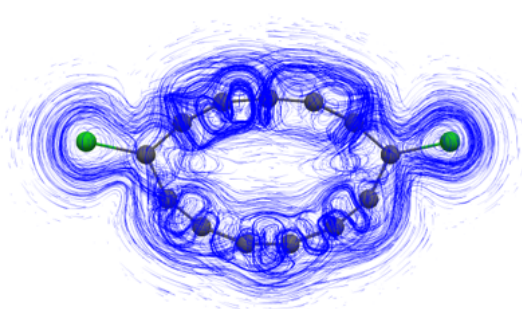

CAS(12 12) – dia

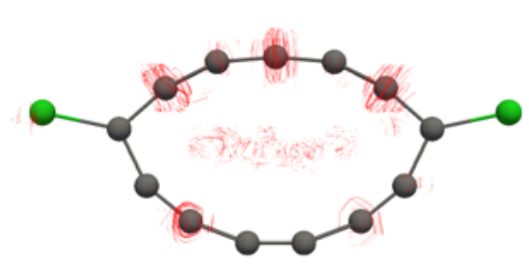

CAS(12 12) – para

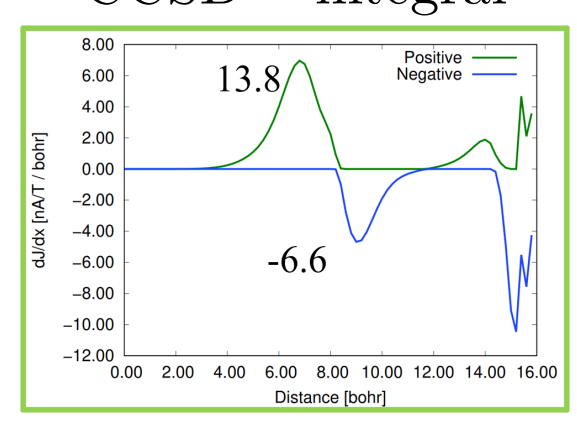

CAS(12 12) – integral

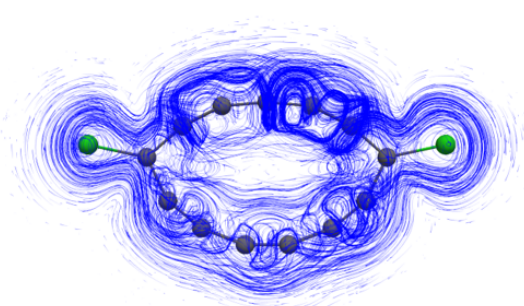

CAS(14 14) – dia

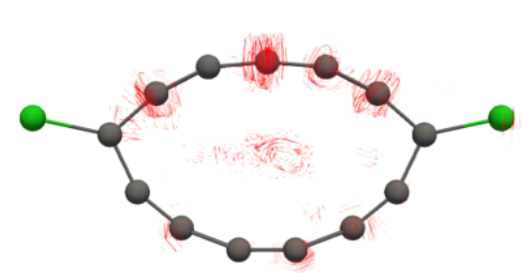

CAS(14 14) – para

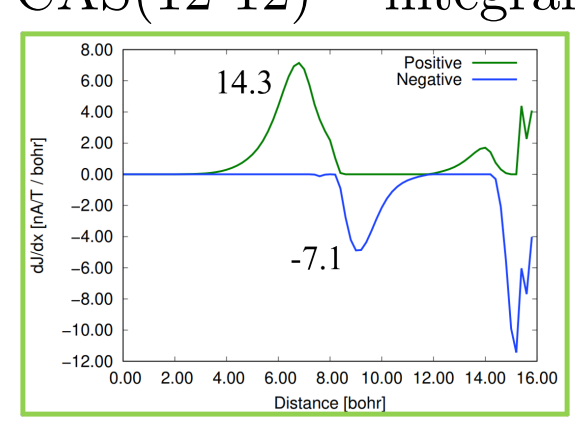

CAS(14 14) – integral

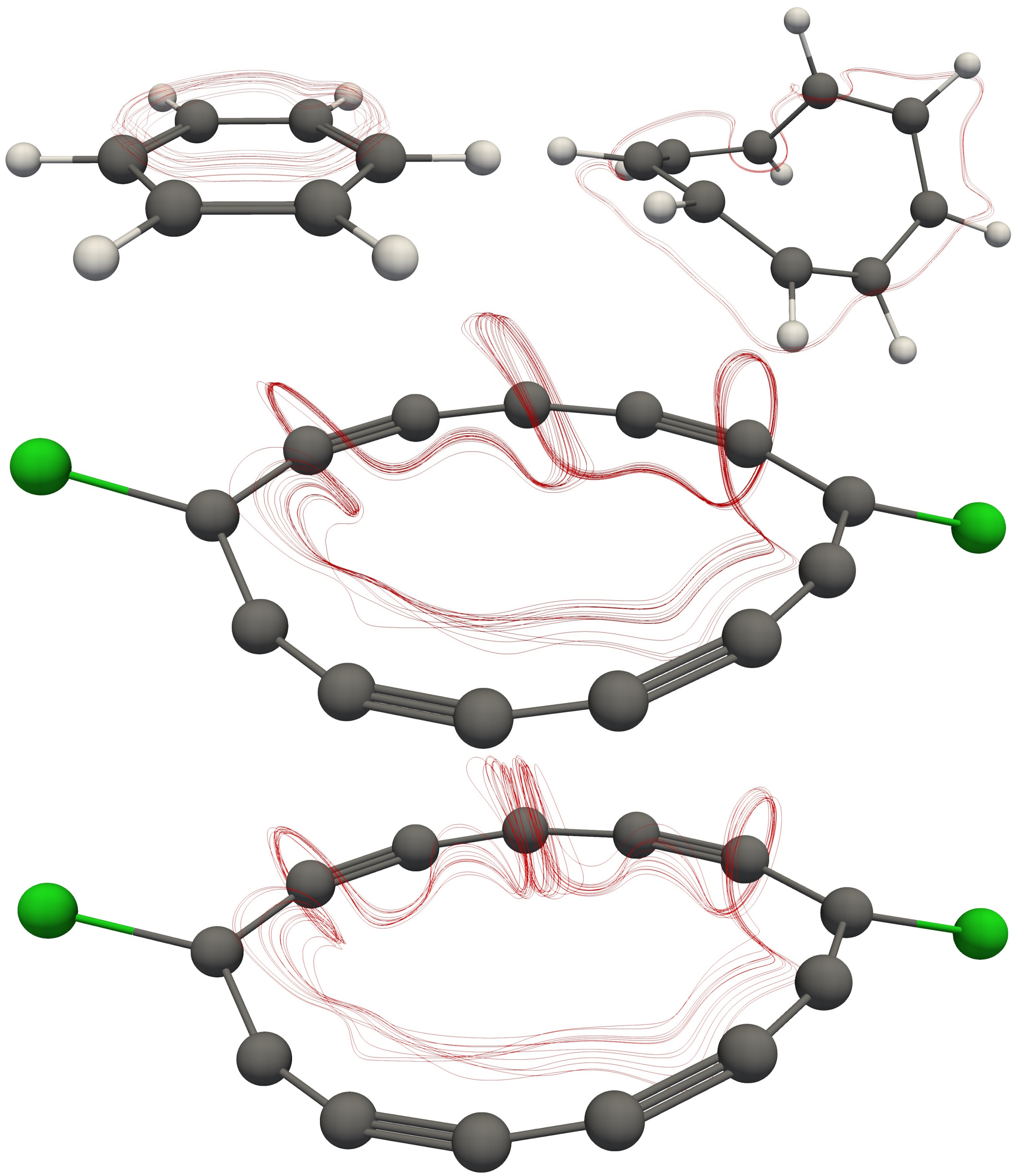

Figure S6: Streamlines of the MIRC of benzene (left top), $C_9H_9^+$ (right top) and $C_{13}Cl_2$ which makes three laps (middle) and four laps (bottom).

Configuration weights

Table S7: The weight of the dominating configuration obtained at the CASSCF level using various active spaces (active electrons,active orbitals) and molecular structures. The molecular structures belonging to the C_{2v} and C_2 point groups are optimized at the ωB97X-D4/def2-TZVP (DFT) and CCSD/def2-TZVP levels of theory.

Active space	DFT-C_{2v}	DFT-C_2	CCSD-C_2
(2,2)	0.97	0.98	0.98
(4,4)	0.93	0.93	0.93
(6,6)	0.91	0.89	0.88
(8,8)	0.88	0.87	0.85
(10,10)	0.86	0.82	0.82
(12,12)	0.76	0.78	0.77

Isotropic shielding constants calculated at the CCSD level

Table S8: The isotropic ^{13}C NMR shielding constants (in ppm) for the C_2 and C_{2v} structures of $C_{13}Cl_2$ calculated at the CCSD level using the molecular structure optimized at the CCSD and ωB97X-D4 (DFT) levels.

Structure	C_1 (C_7)	C_2 (C_6)	C_3 (C_5)	C_4	C_8 (C_{13})	C_9 (C_{12})	C_{10} (C_{11})
C_2(DFT)	115.54	27.17	93.40	54.75	107.96	126.57	128.25
C_2(CCSD)	110.95	26.57	87.66	57.52	103.79	124.77	126.18
C_{2v}(DFT)	164.64	-10.70	161.75	-49.22	110.67	179.72	117.32

Isotropic shielding constants calculated at the CASSCF level

Table S9: The isotropic ^{13}C NMR shielding constants (in ppm) for the C_2 structure of $C_{13}Cl_2$ calculated at the CASSCF level using increasing size of the active space and the DFT molecular structure.

Active space	C_1 (C_7)	C_2 (C_6)	C_3 (C_5)	C_4	C_8 (C_{13})	C_9 (C_{12})	C_{10} (C_{11})
(4,4)	122.5	15.3	75.7	36.9	61.3	128.8	105.6
(6,6)	121.1	15.1	74.5	36.8	77.4	119.8	114.4
(8,8)	108.8	-2.0	80.6	60.7	93.5	112.1	128.4
(12,12)	116.9	36.4	81.8	45.0	90.7	114.8	128.6
(16,16)	116.0	36.8	83.6	50.4	105.6	134.6	114.0

Table S10: The isotropic ^{13}C NMR shielding constants (in ppm) for the C_{2v} structure of $C_{13}Cl_2$ calculated at the CASSCF level using increasing size of the active space and the DFT molecular structure.

Active space	C_1 (C_7)	C_2 (C_6)	C_3 (C_5)	C_4	C_8 (C_{13})	C_9 (C_{12})	C_{10} (C_{11})
(4,4)	190.3	-63.6	192.5	-59.8	-33.8	259.5	79.0
(8,8)	98.5	68.5	94.8	108.4	104.9	81.9	122.1
(12,12)	137.2	-4.7	125.5	57.8	70.9	146.1	119.1

Excitation energies calculated at the XMS-CASPT2 level

Table S11: The excitation energies (in eV) of $C_{13}Cl_2$ calculated at the XMS-CASPT2(8,8) level using the optimized molecular structure of the S_0 state belonging to the C_2 and C_{2v} point groups as well as for the optimized molecular structure of the lowest triplet state (T_1). The molecular structures were optimized at the ωB97X-D4/def2-TZVP level of theory.

State	$C_2(S_0)$	$C_{2v}(S_0)$	$C_1(T_1)$
T_1	0.53	0.34	0.33
S_1	1.25	0.09	0.92
T_2	1.04	0.53	1.33
S_2	1.35	0.50	1.26

Bond lengths

Table S12: Bond lengths (in pm) of the C_2 structure of $C_{13}Cl_2$ calculated at various levels of theory. A Cl atom is substituted to atom C1.

wB97X	CCSD	CASPT2(8,8)	CASSCF(8,8)	TDDFT-MRSF	Bond
132.7	133.3	136.6	131.9	133.8	C1-C2
126.7	127.6	127.4	126.5	126.9	C2-C3
128.1	128.7	131.1	130.3	128.5	C3-C4
141.9	142.9	141.3	143.5	141.0	C1-C13
121.1	121.8	125.7	120.7	122.0	C13-C12
136.7	137.5	135.5	137.4	135.8	C12-C11
121.2	121.8	125.3	120.5	122.1	C11-C10

Cartesian coordinates of the C_2 structure optimized at the ωB97X-D4/def2-TZVP level

```
15
 Energy =   -1415.3486963750
 C   -3.3191230   -0.0634343    0.3282345
```

```
 C   -2.4161672   -0.0638157    1.3005400
 C   -1.2640415   -0.0347114    1.8276985
 C    0.0000000    0.0000000    2.0309604
 C    1.2640415    0.0347114    1.8276985
 C    2.4161672    0.0638157    1.3005400
 C    3.3191230    0.0634343    0.3282345
 C    2.7464458   -0.2452038   -0.9326311
 C    1.8583908   -0.2682728   -1.7558839
 C    0.5999655   -0.0849883   -2.2567769
 C   -0.5999655    0.0849883   -2.2567769
 C   -1.8583908    0.2682728   -1.7558839
 C   -2.7464458    0.2452038   -0.9326311
Cl   -4.9170977   -0.6676281    0.4733387
Cl    4.9170977    0.6676281    0.4733387
```

Cartesian coordinates of the C_{2v} structure optimized at the ωB97X-D4/def2-TZVP level

```
15
 Energy =   -1415.3396587240
 C   -3.4115826    0.0000000    0.2993558
 C   -2.4001489    0.0000000    1.1892794
 C   -1.2779627    0.0000000    1.7634110
 C    0.0000000    0.0000000    1.8702021
 C    1.2779627    0.0000000    1.7634110
 C    2.4001489    0.0000000    1.1892794
 C    3.4115826    0.0000000    0.2993558
 C    2.8127682    0.0000000   -0.9402800
 C    1.8564113    0.0000000   -1.7167143
 C    0.6161685    0.0000000   -2.1884533
 C   -0.6161685    0.0000000   -2.1884533
 C   -1.8564113    0.0000000   -1.7167143
 C   -2.8127682    0.0000000   -0.9402800
Cl   -5.0837437    0.0000000    0.6583003
Cl    5.0837437    0.0000000    0.6583003
```

Cartesian coordinates of the triplet state optimized at the ωB97X-D4/def2-TZVP level without symmetry constraints

```
15
 Energy =   -1415.3562808140
 C   -3.3725927   -0.0099151    0.2569097
 C   -2.3777044   -0.0074947    1.2351406
 C   -1.2502090   -0.0053495    1.7058064
 C    0.0751057   -0.0033143    1.9537110
 C    1.2975858   -0.0001021    1.7875820
```

```
 C    2.5857822    0.0021163    1.4648591
 C    3.3708240    0.0023031    0.3955915
 C    2.6915801    0.0021486   -0.8493082
 C    1.8015382    0.0022410   -1.6701349
 C    0.5566137   -0.0008116   -2.2185970
 C   -0.6583529   -0.0023502   -2.2923086
 C   -1.9267320   -0.0076833   -1.8266016
 C   -2.8465710   -0.0090642   -1.0177156
Cl   -5.0362956   -0.0133117    0.6157146
Cl    5.0894281   -0.0023301    0.4593510
```

Cartesian coordinates of the C_2 structure optimized at the TDDFT-MRSF level

```
15
structure C2
C        2.432281578     -0.071431022     -1.201634624
C       -2.432281578      0.071431022     -1.201634624
C        1.265819925     -0.033133799     -1.700144834
C       -1.265819925      0.033133799     -1.700144834
C        0.000000000      0.000000000     -1.919553079
C        3.337337920     -0.173386943     -0.221466834
C       -3.337337920      0.173386943     -0.221466834
C        2.743339526     -0.397362605      1.037716329
C       -2.743339526      0.397362605      1.037716329
C        1.838665773     -0.357024173      1.854579733
C       -1.838665773      0.357024173      1.854579733
C        0.600328038     -0.110183652      2.352971119
C       -0.600328038      0.110183652      2.352971119
Cl       5.011018823      0.175710414     -0.398838713
Cl      -5.011018823     -0.175710414     -0.398838713
```

Cartesian coordinates of the C_2 structure optimized at the CASSCF(8,8) level using no symmetry

```
15
structure C2
C      3.269148    -0.196454    -0.212494
C      2.426532    -1.206696    -0.223491
C      1.267586    -1.765494    -0.230871
C      0.030514    -2.055277    -0.123210
C     -1.232825    -1.812336     0.083253
C     -2.362498    -1.252079     0.188416
C     -3.263150    -0.290182     0.246122
C     -2.710629     1.010496     0.494815
C     -1.858297     1.863703     0.451353
```

```
C     -0.623996     2.399776     0.174794
C      0.549317     2.415285    -0.098796
C      1.797955     1.917110    -0.383200
C      2.672347     1.088627    -0.450602
Cl     4.900729    -0.304912     0.360320
Cl    -4.915534    -0.4722700   -0.235462
```

Cartesian coordinates of the C_2 structure optimized at the XMC-CASPT2(8,8) level using no symmetry

```
15
structure C2
C       -2.455170882     -1.211833598     -0.029764980
C        2.455630583     -1.214202734      0.033790295
C       -1.276166479     -1.691987826      0.005498035
C        1.276017396     -1.692715359     -0.005536694
C       -0.000194941     -1.991942497     -0.001214105
C       -3.380190226     -0.218603699     -0.180068891
C        3.380387829     -0.220338898      0.183213517
C       -2.791278545      1.025505226     -0.499391872
C        2.791170427      1.022555610      0.504764525
C       -1.840347539      1.840716081     -0.396962529
C        1.841266165      1.838964590      0.397789498
C       -0.610052144      2.346434545     -0.140141949
C        0.611889114      2.346147660      0.138234400
Cl      -5.052482032     -0.374408376      0.195434195
Cl       5.051466011     -0.373239523     -0.198937627
```

Cartesian coordinates of the C_2 structure optimized at the CCSD/def2-TZVP level using the default frozen core approximation of Turbomole

```
15
 Energy =   -1413.2862415247
 C   -3.3219723   -0.0639089    0.3346265
 C   -2.4422373   -0.0696584    1.3358209
 C   -1.2644088   -0.0314349    1.8253671
 C    0.0000000    0.0000000    2.0644236
 C    1.2644088    0.0314349    1.8253671
 C    2.4422373    0.0696584    1.3358209
 C    3.3219723    0.0639089    0.3346265
 C    2.7537968   -0.2727863   -0.9317406
 C    1.8642285   -0.2781266   -1.7632553
 C    0.6012373   -0.0977983   -2.2745092
 C   -0.6012373    0.0977983   -2.2745092
```

```
 C   -1.8642285    0.2781266   -1.7632553
 C   -2.7537968    0.2727863   -0.9317406
Cl   -4.9237565   -0.6865857    0.4414788
Cl    4.9237565    0.6865857    0.4414788
```

Cartesian coordinates of the C_2 structure of $C_9H_9^+$ optimized at the ωB97X-D4/def2-TZVP level of theory

```
18
 Energy =    -348.0234739724
 C   -0.6255262   -1.7432024    0.1872739
 C    0.3965623   -1.6117433   -0.8319372
 C    0.4769363   -0.5656634   -1.6461945
 C   -0.4769363    0.5656634   -1.6461945
 H   -1.2096643    0.5725392   -2.4489076
 C   -0.8752920   -0.9607752    1.2781371
 H    1.0236429   -2.4824109   -0.9993080
 H   -1.2006228   -2.6668101    0.1411667
 C    0.0000000    0.0000000    1.8147981
 H   -1.6377492   -1.3203964    1.9600231
 H    1.2096643   -0.5725392   -2.4489076
 C   -0.3965623    1.6117433   -0.8319372
 H   -1.0236429    2.4824109   -0.9993080
 C    0.8752920    0.9607752    1.2781371
 H    1.6377492    1.3203964    1.9600231
 C    0.6255262    1.7432024    0.1872739
 H    1.2006228    2.6668101    0.1411667
 H    0.0000000    0.0000000    2.9049748
```
